# Supplementary material for: Comparison of the gamma-Pareto convolution with conventional methods of characterising metformin pharmacokinetics in dogs
Source: J Pharmacokinet Pharmacodyn. 2019 Dec 21;47(1):19–45. doi: 10.1007/s10928-019-09666-z (PMC7040082; doi:10.1007/s10928-019-09666-z)
Supplement: Supplementary file 2 — Supplementary material 2 (PDF 152 kb) [file 10928_2019_9666_MOESM2_ESM.pdf]

An incomplete closed form of the gamma-Pareto convolution

Carl A. Wesolowski

This is a proof of Eq. (25) of the text of *Comparison of the gamma-Pareto convolution with conventional methods of characterising metformin pharmacokinetics in dogs.* All of the parameters are non-negative reals, and some of them are definite positive (e.g., β). n is a non-negative integer index and not a parameter. Regions of convergence are not presented and are left to the reader.

Given a gamma-Pareto type I convolution, Eq. (21) of the text,

$$\begin{aligned}\text{GPC}\left(\begin{array}{cc} a & b \\ \alpha & \beta \end{array} \middle| \, t\right) &= \text{GD}(a,b;x) * \text{PD}(\alpha,\beta;x)\ (t) \\ &= \theta(t-\beta)\frac{\alpha b^a \beta^\alpha}{\Gamma(a)}\sum_{n=0}^{\infty}\frac{(-b)^n}{n!}t^{a-\alpha+n-1}B_{1-\frac{\beta}{t}}(a+n,-\alpha)\end{aligned} \quad . \tag{1}$$

For the following identity

$$B_z(p,q) = \frac{z^p}{p}\,{}_2F_1(p, 1-q; p+1; z)\ , \tag{2}$$

we substitute $z = 1-\frac{\beta}{t}, p = a+n, q = -\alpha$ leading to

$$B_{1-\frac{\beta}{t}}(a+n,-\alpha) = \frac{\left(1-\frac{\beta}{t}\right)^{a+n}}{a+n}\,{}_2F_1\left(a+n, \alpha+1; a+n+1; 1-\frac{\beta}{t}\right)\ . \tag{3}$$

Substituting this into Eq. (1)

$$\begin{aligned}&\theta(t-\beta)\frac{\alpha b^a \beta^\alpha}{\Gamma(a)} \\ &\sum_{n=0}^{\infty}\frac{(-b)^n t^{a-\alpha+n-1}\left(1-\frac{\beta}{t}\right)^{a+n}}{n!(a+n)}\,{}_2F_1\left(\alpha+1, a+n; a+n+1; 1-\frac{\beta}{t}\right)\end{aligned} \quad . \tag{4}$$

The Taylor series expansion around ∞ of the summand of which leads to

$$\begin{aligned}&\theta(t-\beta)\frac{\alpha b^a \beta^\alpha}{\Gamma(a)} \\ &\sum_{n=0}^{\infty} t^{a-\alpha+n}\left[-\frac{\pi(-b)^n \csc(\pi\alpha)\Gamma(a+n)}{t\Gamma(\alpha+1)n!\Gamma(a+n-\alpha)} - \frac{(-b)^n\beta^{-\alpha-1}t^\alpha}{n!}\sum_{m=0}^{\infty}\frac{\left(-\frac{\beta}{t}\right)^{m+1}(a-m+n)_m}{m!(\alpha-m)}\right]\ ,\end{aligned} \tag{5}$$

which is the infinite sum of a single term plus a second infinite series, where the second series can be expressed in closed form as a single term

$$\sum_{m=0}^{\infty} -\frac{\left(-\frac{\beta}{t}\right)^{m+1} (a-m+n)_m}{m!(\alpha-m)} = \frac{\beta}{\alpha t} \, {}_2F_1\left(-a-n+1, -\alpha; 1-\alpha; \frac{\beta}{t}\right) \ ,$$

Moreover, from (3), ${}_2F_1(p, 1-q; p+1; z) = \frac{p}{z^p} B_z(p,q)$, if we let $z = \frac{\beta}{t}$, $p = -\alpha$, and $q = a+n$, then

$$ {}_2F_1\left(-a-n+1, -\alpha; 1-\alpha; \frac{\beta}{t}\right) = \alpha \left(\frac{\beta}{t}\right)^{\alpha} B_{\frac{\beta}{t}}(-\alpha, a+n) \ ,$$

which allows to rewrite Eq. (5) as

$$\begin{aligned} &\theta(t-\beta)\frac{\alpha b^a \beta^\alpha}{\Gamma(a)} \\ &\qquad \sum_{n=0}^{\infty} t^{a-\alpha+n} \left[-\frac{\pi(-b)^n \csc(\pi\alpha)\Gamma(a+n)}{t\Gamma(\alpha+1) n! \Gamma(a+n-\alpha)} + \frac{(-b)^n}{t\, n!} B_{\frac{\beta}{t}}(-\alpha, a+n) \right] \end{aligned} \tag{6}$$

Finally, note that the left hand term of this equation simplifies as

$$\begin{aligned} &\theta(t-\beta)\frac{\alpha b^a \beta^\alpha}{\Gamma(a)} \sum_{n=0}^{\infty} t^{a-\alpha+n} \left[-\frac{\pi(-b)^n \csc(\pi\alpha)\Gamma(a+n)}{t\Gamma(\alpha+1) n! \Gamma(a+n-\alpha)} \right] \\ &= -\theta(t-\beta)\pi \csc(\pi\alpha) \frac{b^a \beta^\alpha}{\Gamma(\alpha)} t^{a-\alpha-1} \, {}_1\tilde{F}_1(a, a-\alpha; -bt) \\ &= \theta(t-\beta)\alpha b^a \beta^\alpha \Gamma(-\alpha) t^{a-\alpha-1} \, {}_1\tilde{F}_1(a; a-\alpha; -bt) \ , \end{aligned} \tag{7}$$

where the latter simplification is from $-\pi \csc(\pi\alpha) = \Gamma(-\alpha)\Gamma(\alpha+1) = \alpha\Gamma(-\alpha)\Gamma(\alpha)$. Substituting the Eq. (7) result into Eq. (6) yields

$$\begin{aligned} \mathrm{GPC}\left(\begin{matrix} a & b \\ \alpha & \beta \end{matrix} \,\middle|\, t \right) = &-\theta(t-\beta)\frac{\alpha b^a \beta^\alpha}{\Gamma(a)} t^{a-\alpha-1} \sum_{n=0}^{\infty} \frac{(-b\,t)^n}{n!} B_{\frac{\beta}{t}}(-\alpha, a+n) \\ &+ \theta(t-\beta)\alpha b^a \beta^\alpha \Gamma(-\alpha) t^{a-\alpha-1} \, {}_1\tilde{F}_1(a; a-\alpha; -bt) \ , \end{aligned} \tag{8}$$

which is Eq. (25) of the main text.
