# Supplementary material for: Comparison of the gamma-Pareto convolution with conventional methods of characterising metformin pharmacokinetics in dogs
Source: J Pharmacokinet Pharmacodyn. 2019 Dec 21;47(1):19–45. doi: 10.1007/s10928-019-09666-z (PMC7040082; doi:10.1007/s10928-019-09666-z)
Supplement: Supplementary file 3 — Supplementary material 3 (PDF 306 kb) [file 10928_2019_9666_MOESM3_ESM.pdf]

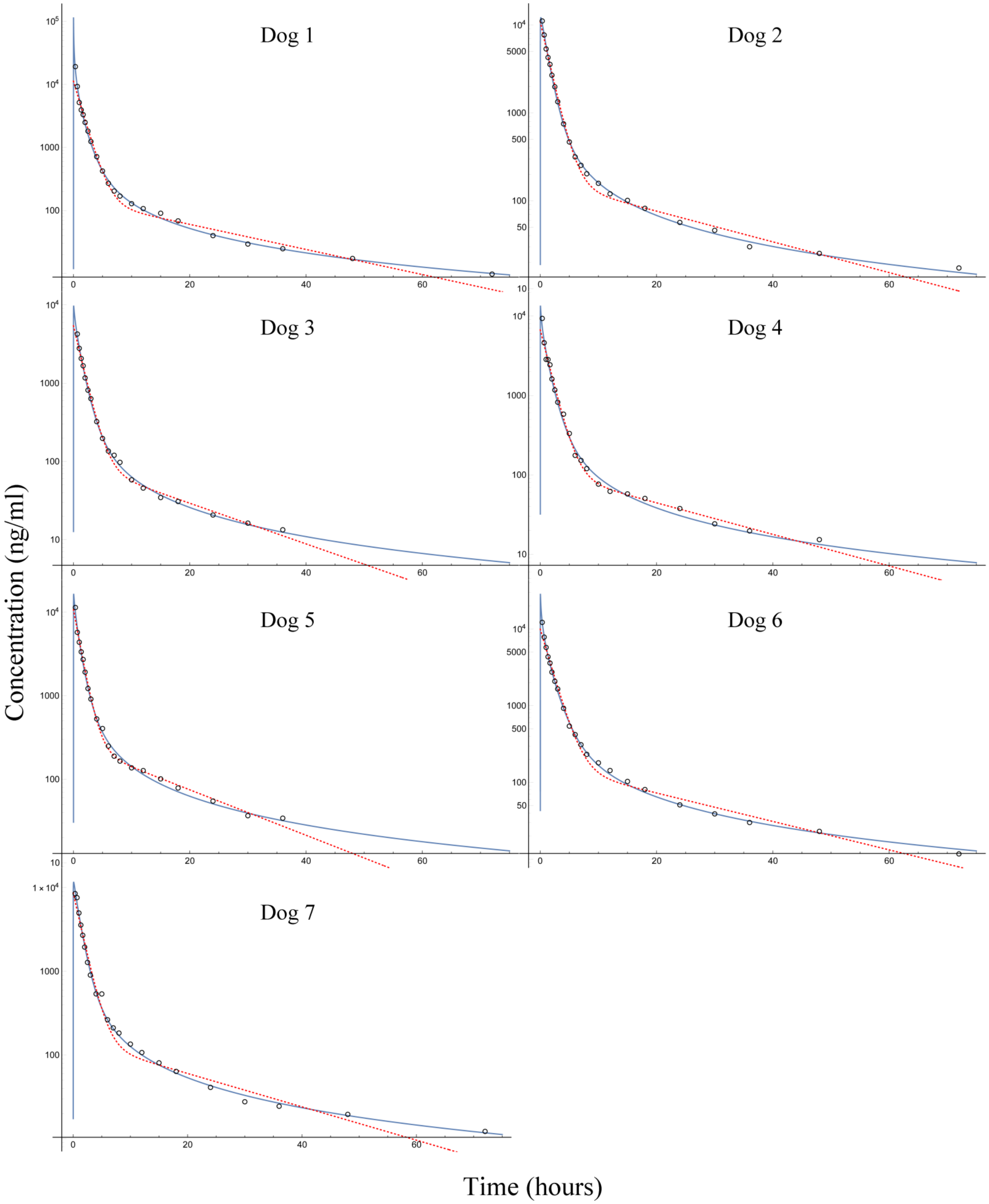

The above shows semi-log plots of fit functions to data. The GPC function is in blue. The earliest portion 25-30 s of that function is zero. A y-axis zero cannot be shown on a semi-log plot. The biexponential fits to the data are shown as dotted red lines, and the data as black circles. Note that the biexponentials underestimated each last sample.
