# Supplementary material for: Comparison of the gamma-Pareto convolution with conventional methods of characterising metformin pharmacokinetics in dogs
Source: J Pharmacokinet Pharmacodyn. 2019 Dec 21;47(1):19–45. doi: 10.1007/s10928-019-09666-z (PMC7040082; doi:10.1007/s10928-019-09666-z)
Supplement: Supplementary file 4 — Supplementary material 4 (PDF 3674 kb) [file 10928_2019_9666_MOESM4_ESM.pdf]

# Comparison of the gamma-Pareto convolution with conventional methods of characterising metformin pharmacokinetics in dogs.

**Carl A. Wesolowski**[†], **Surajith N. Wanasundara**[†], **Paul S. Babyn**[†], **and Jane Alcorn**[‡]

[†] Department of Medical Imaging, Royal University Hospital, College of Medicine

University of Saskatchewan, 103 Hospital Drive, Saskatoon, SK, S7N 0W8, Canada

[‡] College of Pharmacy and Nutrition

University of Saskatchewan, 104 Clinic Place, Saskatoon, SK, S7N 2Z4, Canada

*Corresponding Author: telephone :(306) 665 1515, e-mail: carl.wesolowski@gmail.com

## Abstract

A model was developed for long term metformin tissue retention based upon temporally inclusive models of serum/plasma concentration ($C$) having power function tails called the gamma-Pareto type I convolution (GPC) model and was contrasted with biexponential (E2) and noncompartmental (NC) metformin models. GPC models of $C$ have a peripheral venous first arrival of drug-times parameter, early $C$ peaks and very slow washouts of $C$. The GPC, E2 and NC models were applied to a total of 148 serum samples drawn from 20 min to 72 h following bolus intravenous metformin in seven healthy mongrel dogs. The GPC model was used to calculate area under the curve ($AUC$), clearance ($CL$), and functions of time, $f(t)$, for drug mass remaining ($M$), apparent volume of distribution ($V_d$), as well as $t_{1/2}$ $f(t)$ for $C$, $M$ and $V_d$. The GPC models of $C$ yielded metformin $CL$-values that were 84.8% of total renal plasma flow (RPF) as estimated from meta-analysis. The GPC $CL$-values were significantly less than the corresponding NC and E2 $CL$-values of 104.7% and 123.7% of RPF, respectively. The GPC plasma/serum only model predicted 78.9% drug $M$ average urinary recovery at 72 h; similar to prior human urine drug $M$ collection results. The GPC model $t_{1/2}$ of $M$, $C$ and $V_d$, were asymptotically proportional to elapsed time, with a constant limiting $t_{1/2}$ ratio of $M/C$ averaging 7.0 times, a result in keeping with prior simultaneous $C$ and urine $M$ collection studies and exhibiting a rate of apparent volume growth of $V_d$ that achieved limiting constant values. A simulated constant average drug mass multidosing protocol exhibited increased $V_d$ and $t_{1/2}$ with elapsing time, effects that have been observed experimentally during same-dose multidosing. The GPC heavy-tailed models explained multiple documented phenomena that were unexplained with lighter-tailed models.

**Keywords**: metformin, pharmacokinetics, mathematical modelling pdf, clearance, serum concentration, drug mass, loading dose regimen

## Introduction

Metformin (1,1-dimethylbiguanide), average molecular weight 129.164 g/mol, is a +1 cation at physiological pH with apparent volumes of distribution (dogs) [1] so large that it may be problematic to use classical pharmacokinetic methods to calculate those volumes. Metformin is used among numerous indications as the default first-line treatment for type II diabetes and for cancer chemotherapy in humans. There is a developing interest in treating feline and canine cancer with metformin, which in dogs is more typically used as an anti-hyperglycaemic agent to treat obesity or insulin resistance [1, 2]. In humans, the postulated cancer cell susceptibility to metformin is from defective mitochondrial oxidative phosphorylation and low glucose levels [3]. Metformin is non-metabolised and exclusively plasma-phase renal cleared at an estimated 90%–100% of total renal plasma flow in humans [4]. Seen in association with impaired renal function is dose related lactic acidosis, a rare but serious condition [5–7] suggesting a maximum safe dosing. Also, antineoplastic chemotherapy with metformin potentiates temsirolimus side effects [8], such that secondary metformin effects during therapy are also concerning. For example, extramitochondrial secondary metformin effects are suppression of glycogenolysis, and enhanced cellular glucose uptake relevant to metformin's pharmacodynamics in type II diabetic therapy [9].

| | |
|---|---|
| $AUC$ | Area under the curve |
| $C(t)$ | Concentration (model) |
| CDF | Cumulative density function |
| CI | Confidence Interval, default 95% |
| $C_{in}(t)$ | Concentration, constant infusion |
| $CL$ | Plasma or serum clearance |
| $C_{max}$ | Local peak concentration |
| $C_{\text{obs}}(\mathbf{t})$ | Observed concentrations (vector) |
| $C_{SS}$ | Steady-state concentration, terminal $C_{in}(t)$ |
| CV% | Coefficient of variation |
| $D$ | Dose mass |
| *df* | Degrees of freedom |
| DF(n) | fractional dose multiplier |
| $D_R$ | An acronym of dosing rate |
| E1 | Monoexponential $c\,e^{-\lambda\,t}$ |
| E2 | Biexponential |
| $\text{ED}_n$ | Exponential density of $n$ terms |
| $\text{E}_{\text{PAH}}$ | Renal extraction fraction PAH |
| GD | Gamma distribution |
| GM | Geometric mean |
| GPC | Gamma Pareto distribution |
| H-MRT | Harmonic mean residence time |
| hOCT | Human organic cation transporter |
| IQR | Interquartile range |
| IV | Intravenous |
| $\kappa$ | Equivalent to $AUC$ |
| $\text{L}_2$ | Euclidean norm; vector distance |
| $\text{L}_2^2$ | $(\text{L}_2)^2$, sum of squares, OLS norm |
| $M(t)$ | Drug mass in body (model) |
| MRT | Mean residence time |
| $\mathcal{N}$ | Normal distribution |
| NC | Noncompartmental (analysis) |
| OLS | Ordinary least squares |
| PAH | Para-aminohippuric acid |
| PBPK | Physiologic based pharmacokinetic |
| PD | Pareto distribution |
| pdf | (probability) Density function |
| $R$ | Multiple correlation |
| $r$ | Pearson correlation |
| $rs$ | Rank correlation |
| $\rho_c$ | Lin's concordance correlation |
| RPF | Renal plasma flow |
| RT | Residence time |
| $S(t)$ | Survival function, e.g., $S_{GD}(t)$ |
| SEM | Standard error of the mean |
| SET | Sum of exponential terms |
| $S\tau$ | Mean dose mass during $\tau$ |
| $t_{1/2}(t)$ | Half-life of anything as an $f(t)$ |
| $\tau$ | Dose interval time |
| $\theta(t)$ | Unit step function |
| $V_d(t)$ | Variable apparent volume of distribution |
| $V_d'(t)$ | Rate of change of $V_d(t)$ |
| $V_{d\text{-}area}$ | Terminal apparent volume of distribution |
| $V_{d\text{-}RT}$ | Volume of distribution, a constant from some residence time |
| $V_{H\text{-}MRT}$ | Volume of distribution from H-MRT |
| $V_{SS}$ | Apparent steady-state volume of distribution |

During oral dosing, washed jejunal tissue biopsies had about 30—300× higher metformin

concentrations than that in plasma (human) [10]. Metformin accumulates in the cytosol of erythrocytes, small intestine, skeletal and cardiac muscle cells, hepatocytes, and brain [4, 11–13]. Metformin's primary effect is to suppress gluconeogenesis in mitochondria [14]. Metformin mitochondrial concentration from cultured rat hepatoma cells was 1000× that in plasma at 60 h [15]. Metformin's extra-mitochondrial drug effects appear to occur in other intracellular locations. For example, control of gluconeogenesis was time-delayed using a same-dose regimen [16], and unrelated to blood concentrations [17]. As mitochondria have a very high affinity for metformin, and most eukaryotic cells (except mature erythrocytes) contain many mitochondria, which occupy up to 25 percent of the cytoplasmic volume [18], and are the sites of metformin tumoricidal drug action, with elapsing time one would expect a predominance of body drug mass to be in close proximity to the mitochondrial effector sites.

Intracellular localised metformin effects and plasma drug concentration are kinetically distinct as supported by the observations in rats, dogs and humans that half-lives of drug mass in urine or in erythrocytes are approximately 4.2 to 11.1 times longer than in plasma [4, 13, 19–22]. The difference of half-lives relates to persistent redistribution. To cause persistent redistribution, it would not be enough to add a solitary extra compartment to our model, but with elapsing time even more compartments would be needed. Variable-volume, variable half-life pharmacokinetic modelling is a relatively recent development that allows volume of distribution to be added to a model with elapsing time [23]. The additional concept of a half-life that is not static may seem novel, but was shown to arise in nonequilibrium states (i.e., when the mass half-life is longer than the plasma half-life), and occurs transiently before a compartmental model achieves dynamic equilibrium (i.e., when mass half-life and plasma half-life are equal). Thus, rather than just accept that metformin plasma concentration half-lives are poorly characterised [11], we note that Xie et al. [4] as well as Sambol et al. [24], the latter for oral multidosing, have commented that metformin half-life increased with elapsing time. Indeed, bolus intravenous metformin plasma half-life was 1.5 h as last sampled at 8 h [20], 4.5 h as last sampled at 12 h [22], and 20.4 h, as last sampled at 72 h (dogs) [1], such that there is good evidence that metformin plasma half-lives increase with elapsing time.

To duplicate metformin half-lives of drug mass in urine and erythrocytes that are persistently multiple times longer than in plasma, classical drug models like the sum of exponential terms (SET) and noncompartmental (NC) exponential models are perhaps not the best choices as only a terminal drug mass to plasma concentration half-life ratio of one is possible for those models. SET functions yield transient nonequilibrium models whose time to dynamic equilibrium can be prolonged 7.5 times but not eliminated using gamma distributions with greater than exponential log-convexity enforced using adaptive Tikhonov regulariza-

tion [23, 25, 26]. It is not surprising then that for many bolus drug experiments, a late-time exponential tail underestimates the actual amount of drug remaining in the body [5, 27]. Many intravenously administered drugs has been observed to follow the very heavy-tailed, power-law at late times [28–34]. The statistical form for a power law is the Pareto distribution (PD), which like the Cauchy distribution, has tails so heavy they confer unusual statistical properties and have been given the name *fat*-tailed distributions. Power laws are scale independent and intrinsically fractal. Fractal drug models are consistent with carrier transport [35], and metformin is principally hOCT transported [7]. However, a power-law model does not accurately model the first few hours of concentration [36, 37]. The incorporation of a power function tail into a semi-infinite support model, $0 \leq t < \infty$, was explored with fractional calculus [38], but without consideration of mass conservation and nonequilibrium dynamics. Explored is whether the inclusion of power function tail into a temporally more inclusive model of serum or plasma concentration will yield the permanent nonequilibrium that explains and duplicates the observed persistent mass to concentration ratio of half-lives and whether that forms an accurate enough concentration model to suggest clinical usefulness.

Convolution of a washout model (a monotonic decreasing function) with a gamma distribution (GD) has been used before for correcting the contribution to total residence time of early concentrations [37]. In the current paper, a gamma-Pareto type I convolution (GPC) concentration density function of time was used to model the pharmacokinetics of single bolus intravenous metformin injections is each of seven dogs, most having 22 time-samples collected over 20 min to 72 h. In a GPC model, the PD models the terminal tail of the drug's plasma concentration after the lighter-tailed gamma density (GD) has decayed. Hyperglycaemia therapy with metformin multidosing has shown a progressively increasing effect of decreasing fasting plasma glucose for at least the 8 weeks of testing [39]. Thus, the longer metformin drug mass in tissue may be better linked to drug effect than serum concentration [17], and the modelling introduced should generate accurate drug mass curves from serum data alone, and to be useful should allow for a multidosing regimen that rapidly establishes a constant drug mass in the body, as opposed to dosing for many weeks for that to occur. This may be relevant for cancer chemotherapy as the alternatives are either to wait for months for tissue build up of metformin, which may not provide optimal therapy for rapidly growing tumours, or to give large dosages without knowing when unnecessarily high metformin tissue concentration will occur.

The residual drug masses imply tissue retentions and excretions that were contrasted with published urinary excretion and erythrocyte retention results. The modifications of pharmacokinetic parameters resulting from the application of the new models are presented

and discussed for bolus intravenous metformin disposition curves and body drug mass as functions of time and their half-life functions together with some distinct pharmacodynamic implications arising from them. As some of the results are compared to exponential tailed model results, some statistical characterisation and comparative data analysis of exponential based models is also provided.

## Background

To obtain ratios of half-lives of mass and concentration that do not tend to unity from persistently non-equilibrated models required defining half-life as a function of time. Said ratios naturally arise in the context of variable apparent volume of distribution models, $V_d(t)$. The first mathematically correct $V_d(t)$ models were proposed by Niazi for SET functions [40]. Those models were simultaneously compartmental models and variable volume models, but the interpretations of those different models are distinct. Niazi's work was generalised by some of us for any density function supported on the time is $[0, \infty)$ interval having a varying apparent volume of drug distribution in time, $V_d(t)$, with half-life expressions that vary in time [23]. For variable volume modelling $V_d(t)$ is a drug-o-centric volume of distribution in time. For metformin, the drug is either in the body, or has been eliminated to the urine. We do not know where the $M(t)$ drug mass is in the body. We only know that mass is concentration times volume. That apparent volume of distribution is the imaginary volume that the drug would occupy if it were everywhere at the same concentration as it was observed to be in plasma at that time, and $CL$ eliminates drug mass from that *single volume* no matter how big that volume is.

The general basic equations of variable volume modelling with variable half-life and constant clearance are summarised in Table 1. Note well that these equations are for *bolus* conditions; dose delivered rapidly under intravenous conditions, and do not include dose that remains in any syringe, tubing or vial, nor any depo caused by inadvertent subcutaneous extravasation, i.e., a common, but largely unrecognised problem [41]. Drug mass, $M(t)$, means drug mass in the body, not drug mass excreted. Equations (1)–(2) require clearance $CL$ to be proportional to plasma/serum concentration and constant in time. Strictly speaking, the observed concentrations, $C_{obs}(\mathbf{t})$, for these two equations should be measured where elimination is occurring, which for metformin is where the renal artery contents are cleared in the kidney, and the $C(t)$ model fit to those observations. Note that Eq. (1) reduces to $D = CL\ AUC$ for $t = 0$, where $D = M(t = 0)$ is the dose, $CL$ is plasma/serum clearance and $AUC$ is the area under the curve of $C(t)$ from $t = 0$ to $\infty$. Equation (2) is a mass ratio; the quotient of Eq. (1) evaluated at $t$ divided by its evaluation at $t = 0$, is the

complementary cumulative density function and defines a density function's right-hand tail area (pdf often have two-tails). As a matter of convenience, the complementary cumulative density function is referred to as a survival function $S(t) \stackrel{\text{def}}{=} 1-\text{CDF}(t)$, where CDF(t) is the cumulative density function and $\text{CDF}(t) \stackrel{\text{def}}{=} \int_0^t \text{pdf}(u)du$. This is common practice despite the confusion it causes. That is, a survival function in the strictest sense is discrete, which is not how the term is being used here. Although the name *survival* arose from actuarial usage as the fraction of an initial population surviving in time, here it is the fraction surviving in the body of a unit dose mass at time $t$. Sometimes, survival functions are also used to calculate Relative tail heaviness as per the Appendix Section. Survival functions and CDF are dimensionless (units cancel).

Table 1: Bolus-dose, constant-clearance, general equations

| | | |
|---|---|---|
| Mass as an $f(t)$ | $M(t) = CL\int_t^\infty C(u)\,du$ | (1) |
| Survival function, $S(t)$, definition | $S(t) \stackrel{\text{def}}{=} \int_t^\infty \text{pdf}(u)\,du = \frac{M(t)}{M(0)} = \frac{\int_t^\infty C(u)\,du}{\int_0^\infty C(u)\,du}$ | (2) |
| Conservation of mass | $M(t) = V_d(t)\,C(t)$ | (3) |
| Concentration parsing | $-\frac{C'(t)}{C(t)} = -\frac{M'(t)}{M(t)} + \frac{V_d'(t)}{V_d(t)}$ | (4) |
| Two time-sample exponential half-life | $\xi = -\ln(2)\frac{t_{i+1}-t_i}{\ln(C_{i+1})-\ln(C_i)}$ | (5) |
| Instantaneous half-life | $t_{1/2}; f(t) \stackrel{\text{def}}{=} -\ln(2)\frac{f(t)}{f'(t)}$ | (6) |
| Half-life of drug mass | $t_{1/2}; M(t) = \frac{\ln(2)}{CL}V_d(t)$ | (7) |
| Half-life of concentration | $t_{1/2}; C(t) = \frac{\ln(2)}{CL+V_d'(t)}\,V_d(t)$ | (8) |
| Ratio of $M(t)$ to $C(t)$ half-lives | $\frac{t_{1/2}; M(t)}{t_{1/2}; C(t)} = \frac{CL+V_d'(t)}{CL}$ | (9) |
| Harmonic sum of half-lives | $\frac{1}{t_{1/2}; C(t)} = \frac{1}{t_{1/2}; M(t)} + \frac{1}{-t_{1/2}; V_d(t)}$ | (10) |

Equations (3)–(10) are variable apparent volume of distribution equations and/or variable half-life equations. Of these equations, Eq. (3) and the derivative of its logarithm, parsing of concentration Eq. (4), are the direct results of conservation of mass. For these equations, $V_d(t)$ at $t = 0$ is either undefined for a short time when $C(t)$ models a peripheral venous sample or a $C(t)$ is used that models more proximal concentrations at the injection site itself. Equation (4) relates that the negative relative change in concentration (i.e., a positive number

for decreasing concentration) is the negative relative change of mass (also typically positive) plus the relative drug volume of distribution apparent growth, which latter dilutes/rarefies the relative concentration. For a constant volume of distribution, $V_{d\text{-}RT} = CL\ RT$ can be used, where $RT$ is whichever type of residence time is appropriate to the model under consideration—see the Residence Time Appendix Subsection.

Equation (5) is common knowledge for any two time-samples, $\{t_i, C_i\}$ and $\{t_{i+1}, C_{i+1}\}$. Use of this equation for non-compartmental conditions when $t_{i+1} - t_i$ is small is done with median values from all of the dogs' samples to obtain tractable results—see the Noncompartmental time-sample group, median half-life Appendix Subsection for details. However, Eq. (5) only applies for monoexponential conditions or in the limit if we substitute $f(t) \approx C_i$, and $t_{i+i} = t_i + \Delta t$ and let $\Delta t \to 0^+$, where $f(t)$ is *any* continuously differentiable function. That yields Eq. (6), which is of classical mechanical type, and applies equally well to the evolution of half-life of satellite orbital decay [42] as to variable half-life of concentration of any drug. Half-lives can be both positive and negative. For an $f(t)$ that increases in time, the $t_{1/2}$ is negative. For example, this occurs when concentration is increasing in time in a peripheral vein shortly after intravenous injection. It easier to understand a negative half-life of an increasing function of time when some maximum total value of a measurement is being approached in time by subtracting the measured values from that maximum and viewing the result as a positive half-life of a decreasing function in time, as is done, for example, using total dosage administered for a urinary drug recovery $t_{1/2}$ calculation. However, growth of any function in time, including drug mass accumulating in urine, actually has a negative half-life.

For the half-life of drug mass from the Eq. (6) definition, we substitute Eq. (3) for $f(t)$, and the derivative of Eq. (1) for $f'(t)$, which yields Eq. (7). To find the plasma/serum clearance half-life, Eq. (8), we eliminate the mass terms in concentration parsing Eq. (4) using $M(t) = V_d(t)\, C(t)$ and $M'(t) = -CL\, C(t)$, then rearrange terms to put them into the form of Eq. (6). $V_d'(t)$ is concentration dilution having the same units as $CL$, e.g., ml·min$^{-1}$·kg$^{-1}$. Next, we form the ratio of the mass and concentration half-lives above to yield Eq. (9). We hypothesise that this can have different concentration parsing limits, $V_d'(\infty) = 0$ or $> 0$, [23] and Eq. (33) below, respectively. In the first case, $V_d'(t)$ eventually shrinks to zero, volume dilution of concentration stops, and dynamic equilibrium is achieved. Thereafter, mass half-life is the same as the half-life in plasma/serum. In the second case, $V_d'(\infty)$ is a positive constant and $t_{1/2}; M(t)$ remains longer, e.g., as witnessed as $-t_{1/2}$ of metformin mass in urine, than $t_{1/2}; C(t)$ in plasma/serum. The half-life of $V_d(t)$ adds harmonically, i.e., reciprocally, with mass and concentration half-lives, as follows. Substituting the mass and concentration half-lives, Eqs. (8)–(9), into concentration parsing Eq. (4) yields Eq. (10).

### Constant infusion

Let us assume that elimination rate is proportional to concentration, and that superposition is valid. Using as impulse-response the infinitesimal of an intravenous bolus $C(t)$ model and as constant input of that model, $\theta(t)$, the unit step function in the role of its transfer or *control* function, their convolution is a constant infusion concentration model. Then as $\theta(x) * \text{pdf}(x)\,(t) = \text{CDF}(t)$, where CDF is the cumulative density function,

$$\begin{aligned} C_{in}(t) &= C(x) * \theta(x)\,(t) \\ &= \int_0^t C(x)\,dx = \int_0^\infty C(x)\,dx \int_0^t \text{pdf}\,(x)\,dx \\ &= AUC_{\text{bolus}}\,\text{CDF}\,(t) \equiv C_{SS}\,\text{CDF}\,(t) = \frac{D_R}{CL}\,\text{CDF}(t) \end{aligned} \quad , \tag{11}$$

where the '*in*' of $C_{in}(t)$ is for 'infusion', and where $C_{SS} = \dfrac{D_R}{CL}$ is the steady state concentration [23]. $D_R$, an acronym of *dosing rate*, was used and elsewhere $\text{R}_0$ or $k_0$ are often used. A $\text{CDF}(t)$ has a maximum amplitude of 1 terminally; the occurrence time of a steady state concentration, $C_{SS}$. Thus, to scale $\text{CDF}(t)$, we multiply it by $C_{SS}$ the magnitude of which is $\int_0^\infty C(x)dx$ from the above convolution, such that $C_{SS} \equiv AUC_{\text{bolus}}$. Note that there is no bolus physically, just a result that can predict the magnitude of $C_{in}(t)$ in terms of a previously observed bolus function.

## Methods

This section includes new material such as constant average drug mass multidosing and the explanatory statistical treatment of models as scaled density functions.

## Data collection

The metformin serum concentration data was published elsewhere [1] was reused to reexamine basic modelling assumptions. In that study, seven healthy adult mixed-breed dogs were used including 2 sexually intact males, 2 neutered males, 1 sexually intact female, and 2 spayed females. The dogs were between 2 and 3 years old and weighed between 25.7 and 29.2 kg. The animal study was approved by the University of Saskatchewan's Animal Research Ethics Board, and adhered to the Canadian Council on Animal Care guidelines for humane animal use. Following single intravenous bolus doses of metformin in each of seven dogs, (18.2 mg/kg in dog 1 and 19.5 mg/kg in the other six dogs), 19 to 22 serum samples drawn between 20 min to 72 h were used for modelling—see the Appendix Concentration data and

fitting it, as well as the .xlsx file *data* worksheet in Supplementary Materials 1 for further information.

## Fitting Metformin Concentrations

Area under the curve ($AUC$) inherits its units from those of the curve being used to calculate that area. For example, density functions have a total $AUC_{\text{pdf}}$ of 1, i.e., $\int_0^\infty \text{pdf}(t)\,dt \overset{\text{def}}{=} 1$, with no units (dimensionless) whereas for concentration $AUC_{C(t)}$ has units like $\frac{\text{mg}\cdot\text{h}}{\text{L}}$. The acronym pdf was used for density functions even though the $p$ for *probability* was irrelevant. That is, concentration is not a probability, and a fraction of total $AUC$ per unit time at time $t$ density function, i.e., $\text{pdf}(t) = C(t)/AUC_{C(t)}$ (e.g., units $\text{h}^{-1}$), obeys similar rules to those that would apply to any other density per $x$-axis unit function (pdf) in whatever context the $x$-axis units and $(x, y)$ coordinated values provide. In our case, fitting data with $C(t) = \kappa\ \text{pdf}\,(t)$ yields a concentration curve model, $C(t)$, where $AUC_{C(t)} = \kappa$.

Peripheral venous drug concentration is observed, $C_{obs}(\mathbf{t})$, to be smoothed and delayed by its passage through the circulatory system causing zero initial concentration, $C_{obs}(0) = 0$, to be observed peripherally. In this paper, a virtual washout signal with a $t = 0$ start time, i.e., a gamma distribution, is convolved with a Pareto distribution having a 25—30 s start time to yield a peripheral $C(t)$ model. Ideally, one should minimise bolus error; to not leave drug in the needle track within the dermis by using a large flush bolus injectate to wash the needle and to deliver a high-concentration, low drug volume quickly enough to improve and standardise circulatory mixing [41].

To select disposition curve models one needs to be mindful of which statistical distributions have the best chance of being useful. These selection criteria include inspection of each distribution's parameter types. A distribution's parameter types influence the distribution's derivative fidelity for following the curve shape, and influences the well-posedness of the $AUC$ determination, i.e., whether a unique (global) solution that is not sensitive to noise, called a *stable* solution exists. Stable solutions were obtained in high precision using the Nelder-Mead global optimisation method [43], The practical choice for the modeller is to either find a robust concentration model whose fit to the curve is accurate or to relax the goodness-of-fit criteria, for example by treating AUC as an ill-posed integral of the first kind with use of an inverse method for fitting [25]. Distributions generally take one or more of three types of parameters, those of shape, location and scale. For example, the exponential distribution has a scale parameter, but no shape or location parameters. A location parameter indicates where a distribution is positioned on the $x$-axis. Depending on the distribution, the location may be a best measurement of centrality, but more generally just indicates an $x$-axis posi-

tion. Compared to a distribution without a shape parameter, if we choose a distribution with one or more shape parameters, not only will our goodness of fit generally improve, but the curve shape will be better fitted. Some functions, like the gamma distribution (GD), which has a shape and a scale parameter, may still have an ill-posed $AUC$, such that fitting with Tikhonov regularization rather than ordinary regression may be needed to find a *Tikhonov well-posed* clearance at the price of a small penalty in goodness-of-fit [25].

To fit concentrations we used proportional error minimisation, i.e., $1/C_{obs}^2(\mathbf{t})$ weighted ordinary least squares fitting of models to data [37, 44–47]—see the Concentration data and fitting it Subsection of the Appendix for details on this and on proportional assay error, how to quantify root mean square proportional error (rrms), and noncompartmental methods. The GPC function was the experimental model fit to data. It is calculated from hundreds of sums using 65 decimal place arithmetic to mitigate roundoff error. The GPC algorithm with Nelder-Mead regression was designed for precision and accuracy to converge to 30 decimal places. The GPC algorithm used, Eq. (25) below, purpose written in Mathematica 12.0.0.0 code, was not optimised for speed such that the run time for each case was $34 \pm 12$ min on a 2.7 GHz Intel Core i7 CPU using four parallel processors. In *post hoc* testing this was reduced to 28 min using series acceleration methods, i.e., not much faster and that despite reducing the call to the GPC algorithm from 45 ms to 19 ms average. Thus, much of the elapsed time for regression is due to factors other than GPC algorithm run time, for example performing constrained rather than unconstrained regression and the use of extended precision. Individual subject parameter statistics (CV%) are not a feature of the global optimisation search method used, and see the Parameter errors from model-based bootstrap Appendix Section for a computationally intensive analysis of algorithmic robustness. For E2, parameter CV% are typically calculated from local optimisation gradients, e.g., using the Levenberg-Marquardt method and similar methods, which may not be the CV% from least error, i.e., global, solutions, and only indirectly examine for robustness. Sample-time groups are used in the text, especially in Noncompartmental time-sample group, median half-life. These are merely groups of samples having in common the same times following bolus injection that were used for drawing samples from all of the subjects.

## Dose regimen models

The simplest dose regimen is repeat constant dosing. Another dose regimen of possible interest for vasoactive drugs, e.g., dipyridamole, would be to keep the mean plasma drug concentration constant for each dose interval. A dose regimen for drugs like metformin that have major effects in cytosol and mitochondria *and* that accumulate in cytosol, may have as

a goal to dose for the same mean drug mass retained in the body during each dose interval.

Let us assume first order elimination and superposition. Then, a protocol for keeping a constant mean unit dose drug mass in the body starts with a unit intravenous dose from the identity function for a density, $\int_0^\infty \text{pdf}(t)\,dt = 1$, break it into dose periods of $\tau$, and divide that series by $\tau$ to calculate the average fraction of a unit dose mass per unit time during each dosing interval, which is the average mass over the dose interval, which written from the survival function equals $\Delta S(t)/\tau$, i.e., $S\tau(i) = \frac{1}{\tau}\,\{S[\tau(i-1)] - S(\tau i)\}$, for $i = 1, 2, 3 \ldots$.

$$\frac{1}{\tau} = \frac{1}{\tau}\int_0^\tau \text{pdf}(t)dt + \frac{1}{\tau}\int_\tau^{2\tau} \text{pdf}(t)dt + \frac{1}{\tau}\int_{2\tau}^{3\tau} \text{pdf}(t)dt \; + \; \ldots \tag{12a}$$

$$= \quad S\tau(1) \quad + \quad S\tau(2) \quad + \quad S\tau(3) \; + \; \ldots \quad . \tag{12b}$$

During the first dose interval of duration $\tau$, to augment the dosage to yield an average drug mass during $\tau$ to be a unit dose, the dose administered is augmented by multiplying the unit dose by the reciprocals of the first terms of right sides Eq. (12a) and (12b) to yield the dose factor, $DF$

$$DF(1) = \frac{\tau}{\int_0^\tau \text{pdf}\,(t)\,dt} = \frac{1}{S\tau(1)} \geq 1 \quad . \tag{13}$$

To obtain a dose factor for a constant average unit dose mass during the second $\tau$ interval one includes the contribution from the first dose second dose interval, and the second dose first dose interval, which simplifies to yield

$$DF(2) = DF(1)\,[1 - DF(1)\;S\tau(2)] \quad .$$

For $n \geq 2$, this equation generalises to be the recursion,

$$DF(n) = DF(1)\left[1 - \sum_{i=1}^{n-1} DF(i)\,S\tau(n+1-i)\right] . \tag{14}$$

Note that as $n \to \infty$, $DF(n)$ goes to that fractional dose multiplier, $> 0$, that allows an average of a unit dose to be maintained in the body during the $n^{\text{th}}$ dose interval. A word of caution, for accurate results from Eq. (14), the terminal tail of the model used must accurately match plasma/serum drug concentrations.

## Distributions

This subsection presents the distributions (pdf) used. Some properties of these distributions are: The convolution of two pdf is a pdf. The $AUC$ of a pdf is 1. $C(t) = \kappa\,\text{pdf}$ where $\kappa$ is the

$AUC$ of $C(t)$. A survival function, $S(t)$, is the $t$ to $\infty$ integral of the pdf. For intravenous bolus conditions, regardless of distribution type, when $t = 0$, $S(0) = 1$, and the entire dose fraction is within the body.

### Gamma distribution

The gamma distribution (GD) is given by

$$\text{GD}(a, b; t) = \frac{1}{t} \, \frac{e^{-b\,t}(b\,t)^a}{\Gamma(a)} \, \theta(t) \; , \tag{15}$$

where the gamma function satisfies $\Gamma(a) = \int_0^\infty e^{-t} t^{a-1} \, dt$, and $\theta(t)$ is the unit step function, i.e., 0 for $t < 0$ and 1 for $t \geq 0$. The GD is an exponential density (ED, below) when $a = 1$. The GD also has a $(+\infty)$ discontinuity at $t = 0$ when $0 < a < 1$. However, that discontinuity is integrable with zero area in the limit as $t \to 0$. Thus, the survival function ($S_{\text{GD}}$) is defined at $t = 0$, and,

$$S_{\text{GD}}(a, b; t) = \frac{\Gamma(a, b\,t)}{\Gamma(a)} \, \theta(t) = Q(a, b\,t) \, \theta(t) \; , \tag{16}$$

where the upper incomplete gamma function satisfies $\Gamma(a, z) = \int_z^\infty u^{a-1} e^{-u} du$, and $Q(a, z)$ is its regularised form. The GD rate parameter is $b$, whereas $\frac{1}{b}$ is the scale parameter. The shape parameter for the GD is $a$. The shape parameter aids in fitting disposition curves and their shapes. There is no location parameter.

### Pareto distribution

Negative power functions of time have an undefined $(+\infty)$ discontinuity at $t = 0$ that is not integrable $(+\infty)$. Thus, a power function as a density function could have a minimum positive start time, $\beta > 0$, thereby allowing for integration that avoids the discontinuity. Just such a function is the type I Pareto distribution (PD),

$$\text{PD}(\alpha, \beta; t) = \frac{\alpha}{t} \left( \frac{\beta}{t} \right)^{\alpha} \theta(t - \beta) \; . \tag{17}$$

Note the shift to the right of the unit step function; $\theta(t - \beta)$, i.e., the PD function is zero for $t < \beta$. The PD survival function ($S_{\text{PD}}$) is,

$$S_{\text{PD}}(\alpha, \beta; t) = \begin{cases} \left( \dfrac{\beta}{t} \right)^{\alpha} & t \geq \beta \\ 1 & 0 \leq t < \beta \\ 0 & t < 0 \end{cases} \; . \tag{18}$$

$\beta$ is the scale parameter. The shape parameter is $\alpha$. There is no location parameter.

### Sums of exponential term distribution

Sums of exponential terms (SET) models are equivalently $C_{\text{SET}}(t) = \theta(t)\ \sum_{i=1}^{n} c_i\, e^{-\lambda_i\, t}$, where $n$ is any positive integer including 1, and without loss of generality $\lambda_1 \geq \lambda_2 \geq \lambda_3 ... \geq \lambda_n > 0$. SET models have been incorrectly criticised as having meaningless $c_i$ coefficients. In a statistical context note that $\lambda e^{-\lambda t}$ is an exponential density, then let $\sum_{i=1}^{n} p_i = 1$, where the $p_i$ (not probabilities here) are the fractions of the model's total density attributed to each exponential density term. Then,

$$\text{ED}_n(t) = \ \theta(t)\ \sum_{i=1}^{n} p_i\, \lambda_i\, e^{-\lambda_i\, t}, \tag{19}$$

where the $\lambda_i$ decay coefficients are rate (i.e., 1/scale) parameters. The $c_i$ of SET functions relate to the $\text{ED}_n$ parameters as $c_i = \kappa\, \lambda_i\, p_i$, where concentration is $C_{\text{SET}}(t) = \kappa\, \text{ED}_n$ in which $\kappa = \int_0^\infty C_{\text{SET}}(t)dt = AUC(C_{\text{SET}})$. Note that a scaled monoexponential, an E1, is not a monoexponential density, ED, as E1$= AUC_{\text{E1}}$ ED. So when we refer to a biexponential we write E2, where a biexponential density is an $\text{ED}_2$. The survival function is

$$S_{\text{ED}n} = \ \theta(t)\ \sum_{i=1}^{n} p_i\, e^{-\lambda_i\, t}\,. \tag{20}$$

Note that $\text{ED}_n$, $S_{\text{ED}n}$ and SET only have scale parameters (or their reciprocals). There are no shape parameters to aid for fitting curve shapes or for extrapolation, and the solutions for $\text{SET}_{n\geq 2}$ lack robustness. For example, biexponentials (E2) solved for 4 time-samples sometimes have solutions as $\in \mathbb{C}$, not $\in \mathbb{R}$ [36]. There are no location parameters.

### The gamma-Pareto convolution distribution, basic formulas

Derived from series expansion of the GD exponential, Eq. (15), followed by convolution with a PD, Eq. (17) and summation of the expanded parts,[1] the GPC model inherits two shape parameters, $a$ and $\alpha$, and two scale parameters, $b$ and $\beta$ from its convolved pdf's,

$$\begin{aligned} \text{GPC}\left( \begin{array}{cc} a & b \\ \alpha & \beta \end{array} \middle|\, t \right) &= \text{GD}(a,b;x) * \text{PD}(\alpha,\beta;x)\ (t) \\ &= \theta(t-\beta)\frac{\alpha b^a \beta^\alpha}{\Gamma(a)} \sum_{n=0}^{\infty} \frac{1}{n!}(-1)^n b^n t^{a-\alpha+n-1} B_{1-\frac{\beta}{t}}(a+n,-\alpha) \end{aligned}\ , \tag{21}$$

[1]This is parallel to the development of the type II Pareto, GPC model [48].

where the incomplete beta function is $B_z(a,b) = \int_0^z u^{a-1}(1-u)^{b-1}du$. The GPC function has a concentration start time, $\beta > 0$, and a circulatory mixing start time of 0. In practice, GPC models chain, i.e., convolve, two independent washout functions (monotonic decreasing; GD iff $a \leq 1$ and a PD) that only when combined produce a circulatory peak concentration. The GD washout models relate to the circulatory mixing that starts at $t = 0$, whereas the PD models relate to the injection to sampling venous-venous delay of a few dozen seconds ($= \beta$) and the terminal rate of the increase in apparent volume of distribution in time [$CL/\alpha$, Eq. (33), below].

For use with $M(t)$, and other functions, we integrate the GPC function from 0 to $t$ to construct its cumulative density function (proof by differentiation),

$$\begin{aligned} &\text{CDF}_{\text{GPC}}\left(\begin{array}{cc} a & b \\ \alpha & \beta \end{array}\,\middle|\, t\right) = \theta(t-\beta)\frac{\alpha\beta^{\alpha}b^{a}}{\Gamma(a)} \times \\ &\sum_{n=0}^{\infty}\frac{(-1)^n b^n}{n!(a-\alpha+n)}\left\{\begin{array}{l} \left(t^{a-\alpha+n}-\beta^{a-\alpha+n}\right)B_{1-\frac{\beta}{t}}(a+n,-\alpha) \\ \quad -\beta^{a-\alpha+n}\left[\frac{\left(\frac{t}{\beta}-1\right)^{a+n}}{a+n}+B_{\frac{\beta}{t}}(-\alpha,a+n)-B(-\alpha,a+n)\right] \end{array}\right\}, \end{aligned} \tag{22}$$

where, for $z = 1$, $B_z(a,\,b)$ becomes the beta function $B(a,b) = \frac{\Gamma(a)\,\Gamma(b)}{\Gamma(a+b)}$. As it is needed for the half-life of concentration, we take the derivative of the GPC,

$$\begin{aligned} &\text{GPC}'\left(\begin{array}{cc} a & b \\ \alpha & \beta \end{array}\,\middle|\, t\right) = \theta(t-\beta)\frac{\alpha\beta^{\alpha}b^{a}}{\Gamma(a)} \times \\ &\sum_{n=0}^{\infty}\frac{(-b)^n}{n!}t^{-1}\left[(a-\alpha+n-1)t^{a-\alpha+n-1}B_{1-\frac{\beta}{t}}(a+n,-\alpha)+\beta^{-\alpha}(t-\beta)^{a+n-1}\right] \end{aligned} . \tag{23}$$

**Asymptotes of the gamma-Pareto convolution**

Two asymptotes to the GPC function were identified for $t$ is a sufficiently long time. The first is the Pareto Type I distribution itself, and is the desired result; to write a power function tail into a more general PK model,

$$\text{GPC}\left(\begin{array}{cc} a & b \\ \alpha & \beta \end{array}\,\middle|\, t\right) \sim \alpha\beta^{\alpha}t^{-1-\alpha}\,, \tag{24}$$

and is a straight line on a log-log plot, where ($\sim$) means asymptotic to. This occurs because the GD tail decays quickly and the PD tail decays very slowly, see Relative tail heaviness.

The second GPC asymptote was obtained by Taylor series expansion of Eq. (21), with the proof appearing as the .pdf file labelled Supplementary Materials 2,

$$\mathrm{GPC}\left(\begin{matrix} a & b \\ \alpha & \beta \end{matrix}\,\middle|\, t\right) = \begin{array}{l} -\theta(t-\beta)\dfrac{\alpha b^a \beta^\alpha}{\Gamma(a)} t^{a-\alpha-1} \displaystyle\sum_{n=0}^{\infty} \frac{(-b\,t)^n}{n!} B_{\frac{\beta}{t}}(-\alpha, a+n) \\ \quad - \theta(t-\beta)\pi \csc(\pi\alpha)\dfrac{b^a \beta^\alpha}{\Gamma(\alpha)} t^{a-\alpha-1}\, {}_1\tilde{F}_1(a, a-\alpha; -bt) \end{array}, \tag{25}$$

where ${}_1\tilde{F}_1(a;b;z) \overset{\text{def}}{=} \Gamma(b)\sum_{k=0}^{\infty} \frac{(a)_k}{(b)_k}\frac{z^k}{k!}$ is the regularised form of Kummer's confluent hypergeometric function of the first kind wherein $(a)_k$ and $(b)_k$ are Pochhammer symbols, i.e., generalised ascending factorials, $(a)_k = a(a+1)\dots(a+k-1) = \Gamma(a+k)/\Gamma(a)$. The closed form term (beginning $-\pi$) of Eq. (25) is positive when $0 < \alpha < 1$ and $b\,t$ are sufficiently large, while the limit of the sum converges to zero. This forms a second GPC asymptote that approaches the GPC more rapidly than the PD tail, and finds use for computing the GPC when $t$ is long, e.g., approximately $> 100$ h. That is, for $0 < \alpha < 1$ and $t$ sufficiently long, that term and the GCP are asymptotic to each other,

$$\mathrm{GPC}\left(\begin{matrix} a & b \\ \alpha & \beta \end{matrix}\,\middle|\, t\right) \sim -\pi \csc(\pi\alpha)\frac{b^a \beta^\alpha}{\Gamma(\alpha)} t^{a-\alpha-1}\, {}_1\tilde{F}_1(a, a-\alpha; -bt)\ . \tag{26}$$

## What the concentration models imply

### Drug mass

The drug mass remaining in the body, is from the GPC CDF, Eq. (22) applied to Eq. (2),

$$M(t) = D\, S_{\mathrm{GPC}} = D\left[1 - \mathrm{CDF}_{\mathrm{GPC}}\left(\begin{matrix} a & b \\ \alpha & \beta \end{matrix}\,\middle|\, t\right)\right]\ , \tag{27}$$

where $M(0) = D$ is the dose.

### Half-life functions

The half-life function of metformin model concentration, $t_{1/2}; C(t)$, as observed in a peripheral venous sampling site, comes from substituting the GPC$(t)$, Eq. (21), and its derivative Eq. (23) for the $f(t)$ and $f'(t)$ of Eq. (6),

$$t_{1/2}; C(t) = -\ln(2)\, \frac{\mathrm{GPC}\left(\begin{matrix} a & b \\ \alpha & \beta \end{matrix}\,\middle|\, t\right)}{\mathrm{GPC}\,'\left(\begin{matrix} a & b \\ \alpha & \beta \end{matrix}\,\middle|\, t\right)}\ . \tag{28}$$

Next, the half-life of drug mass remaining in the body from Eqs. (2), (22), (21), and (6) is

$$t_{1/2}; M(t) = \ln(2) \frac{1 - \text{CDF}_{\text{GPC}}\left( \begin{matrix} a & b \\ \alpha & \beta \end{matrix} \middle| t \right)}{\text{GPC}\left( \begin{matrix} a & b \\ \alpha & \beta \end{matrix} \middle| t \right)} \; . \tag{29}$$

The asymptotic half-life of the GPC concentration model was obtained by substitution of the first GPC asymptote, Eq. (24), and its derivative into half-life Eq. (6), and is linear

$$t_{1/2}; C(t) \sim \frac{\ln(2)}{\alpha + 1}\, t \; ; \qquad t \to \infty \; . \tag{30}$$

Similarly, the half-life of drug mass is asymptotic to

$$t_{1/2}; M(t) \sim \frac{\ln(2)}{\alpha}\, t \; ; \qquad t \to \infty \; , \tag{31}$$

a linear function of time. Equations (30) & (31) suggest that metformin GPC half-lives can be any time, no matter how long, just by measuring that half-life at a correspondingly late time. From Eqs. (31) divided by (30) the terminal ratio of half-lives of drug mass remaining in the body to serum concentration is

$$\frac{t_{1/2}; M(t)}{t_{1/2}; C(t)} \sim \frac{\alpha + 1}{\alpha}; \qquad t \to \infty \tag{32}$$

which as $\alpha > 0$, is $> 1$. That this limit is not equal to 1, as it would be for lighter-tailed models, exemplifies why Pareto distribution tails have been called *fat*-tailed historically; because of their provocatively different properties from those of lighter-tailed distributions.[2] There are now two expressions for the mass to concentration ratio, one of them for any variable volume model, Eq. (9), and the other from the PD asymptote to the GPC, Eq. (24). Combining these expressions yields the terminal rate for volume of distribution apparent growth for our fat-tailed model of $V_d'(\infty)$,

$$V_d'(t) \sim \frac{CL}{\alpha}; \;\; t \to \infty \; . \tag{33}$$

As $\alpha$ is dimensionless, $V_d'$; the rate of growth of $V_d$, has the same units as $CL$. Rewriting of the half-life harmonic sum Eq. (10) and solving for the asymptote of $t_{1/2}; V_d(t)$ yields its negative half-life (growth)

$$-\, t_{1/2}; V_d(t) \sim \ln(2)\, t \; ; \;\; t \to \infty \; . \tag{34}$$

[2]Ironically, the fat-tailed distribution of wealth inspired Karl Marx's [49], as well as Benito Mussolini's economic policies as diametrically opposite and extreme reactions to the same statistics [50].

## Results

The gamma-Pareto convolution (GPC) distributions' parameters for metformin in seven dogs appear in Table 2. As a global search routine, the Nelder-Mead method does not generate case-wise CV% results. For that information, please refer to the Parameter errors from model-based bootstrap Appendix section. Figure 1 shows GPC models fit to the data over four orders of magnitude of serum concentration of metformin and 72 h of data sampling for 7 subjects. Also shown as straight lines on the same log-log plots are the GPC's Pareto power-function asymptotes, Eq. (24). A back extrapolated GPC concentration model is shown in Figure 2, with zero initial concentration that cannot be shown in the Figure 1 log-log plots. For semi-log plots please see the .pdf file labelled Supplementary Materials 3. Goodness of fit was shown as relative error plots of residuals of fitting in Figure 3 for the GPC models and Figure 4 for biexponential (E2) models. Recall (Concentration data and fitting it) that relative errors are identically $1/C^2$ weighted residuals, and note that the sample-times were approximately equally logarithm of time distributed. Regression in $y$ does not see the $x$-axis spacing, thus the equal spacing between grouped sample-times on these plots.

From Table 2 the GPC models mimicked experimental concentrations and achieved an early concentration peak ranging from 0.66 min to 6.17 min total elapsed time. GPC shape

Table 2: Shown are parameters from gamma densities (GD), Pareto densities (PD) and both from Gamma-Pareto convolution (GPC) fitting of concentrations data for 7 dogs.

| | GD | | PD | | GPC | | | | | |
|---|---|---|---|---|---|---|---|---|---|---|
| Dog | $a$ | $b$ | $\alpha$ | $\beta$ | $AUC$ | Peak | $V'_d(\infty)$ | $CL$ | $H\text{-}MRT$ | $V_{H\text{-}MRT}$ |
| * | (none) | (per h) | (none) | (s) | ($\frac{\text{mg}\cdot\text{h}}{\text{L}}$) | (min) | ($\frac{\text{ml}}{\text{min}\cdot\text{kg}}$) | ($\frac{\text{ml}}{\text{min}\cdot\text{kg}}$) | (h) | ($\frac{\text{L}}{\text{kg}}$) |
| 1 | 0.3493 | 0.7318 | 0.2644 | 25 | 31.16 | 0.66 | 36.9 | 9.8 | 0.118 | 0.069 |
| 2 | 0.8112 | 0.9993 | 0.1365 | 25 | 28.18 | 6.17 | 84.5 | 11.5 | 0.557 | 0.386 |
| 3 | 0.6689 | 0.9107 | 0.2010 | 25 | 12.15 | 2.21 | 133.0 | 26.7 | 0.322 | 0.516 |
| 4 | 0.6092 | 0.8062 | 0.1726 | 25 | 16.73 | 1.70 | 112.5 | 19.4 | 0.332 | 0.387 |
| 5 | 0.6435 | 1.1035 | 0.1199 | 25 | 26.21 | 2.21 | 103.4 | 12.4 | 0.413 | 0.307 |
| 6 | 0.5194 | 0.6137 | 0.1929 | 30 | 28.43 | 1.34 | 59.3 | 11.4 | 0.300 | 0.206 |
| 7 | 0.7629 | 1.0518 | 0.1571 | 30 | 22.10 | 4.85 | 93.6 | 14.7 | 0.477 | 0.421 |
| mean | 0.6235 | 0.8882 | 0.1778 | 26 | 23.57 | 2.73 | 89.0 | 15.14 | 0.360 | 0.327 |
| SD | 0.1546 | 0.1791 | 0.0479 | † | 6.94 | 2.01 | 32.5 | 6.01 | 0.141 | 0.149 |
| CV% | 24.8 | 20.2 | 27.0 | — | 29.4 | 73.4 | 36.5 | 39.7 | 39.3 | 45.5 |

*Units row: *None* means *dimensionless*. Peak time is at the maximum of the GPC model. $V'_d(\infty)$ is the GPC model, terminal rate of apparent growth of $V_d$. *H-MRT* is harmonic mean residence time. $V_{H\text{-}MRT}$ is the constant volume of distribution from the *H-MRT*. SD is standard deviation. CV is coefficient of variation.

†As $\beta$ is constrained, its SD and CV are not meaningful.

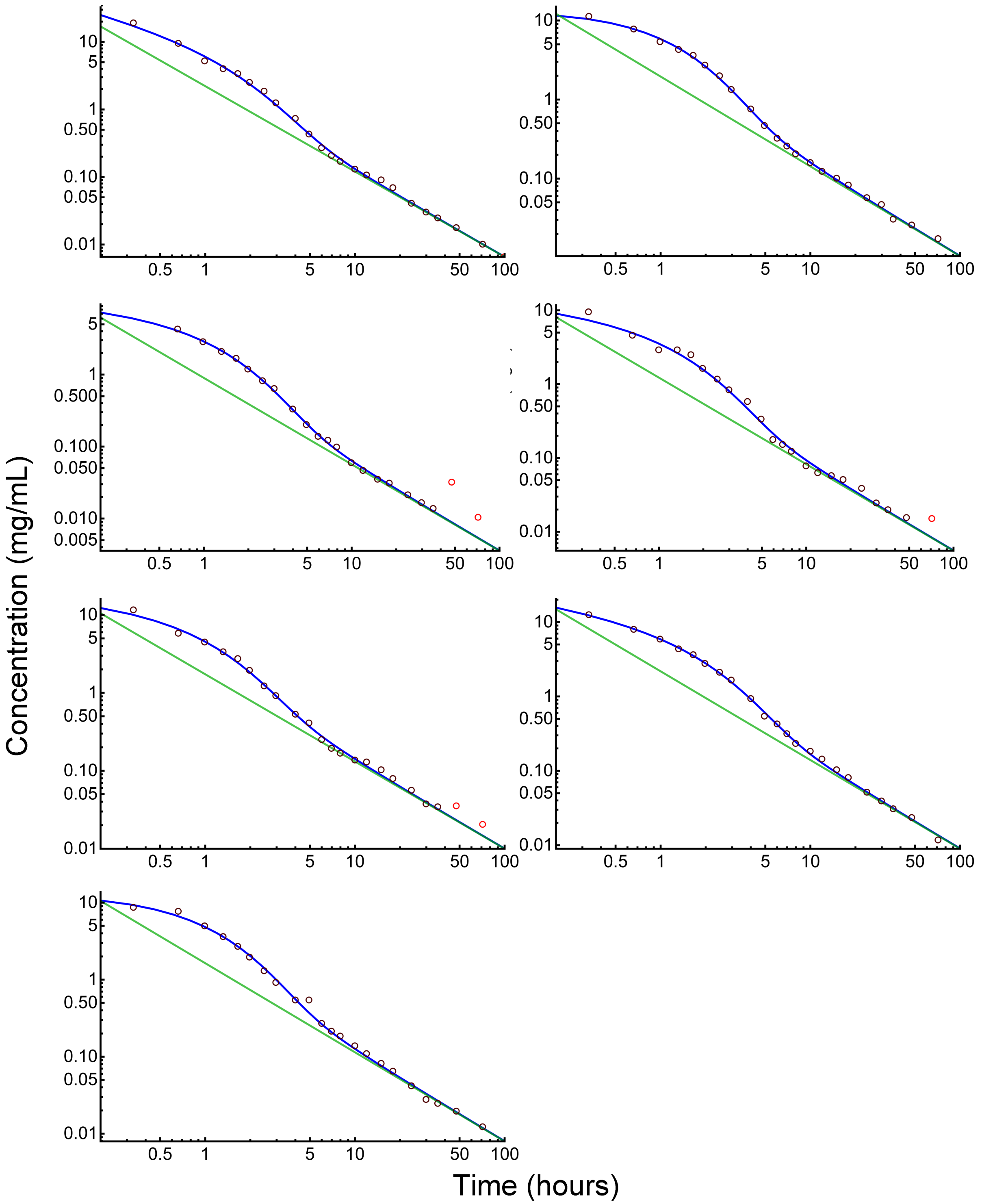


Figure 1: Log-log profiles of metformin concentrations versus time in 7 dogs. The black circles are data. The red circles are the five samples left out following outlier testing. The blue curves are the GPC concentration models and the green lines are the Pareto (power function) asymptotes of the GPC models. Note the maximum distance between the blue and green curves at 1-2 hrs. That is the peak effect time of GD convolution. At those times, the convolution of GD function's slow mixing increased the PD function magnitude several fold.

Figure 2: This shows the early time pdf, i.e., each having an $AUC$ of 1, of the GPC model (blue) and the gamma density (GD, in red) for dog 5. The GD is shown truncated at its top, and is the faster decaying of two GPC constituent functions. Note that the GPC starts a small time (25 s here) later than the GD, and the GD is less than the GPC for late time.

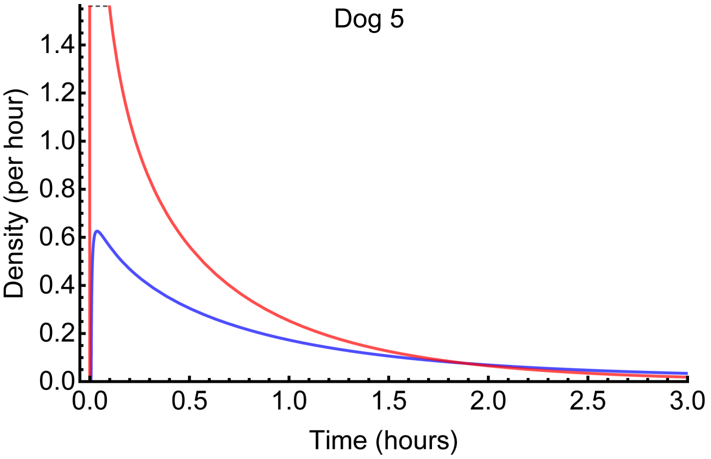


parameters from the GD were $a < 1$ for which there were infinite concentrations at $t = 0$. These GD curves are *washout* or monotonic decreasing curves with a rate (1/scale) parameter, $b \approx 1$ h$^{-1}$. The GPC PD parameters are $\alpha$ and $\beta$. The PD shape parameters, $\alpha$, in the power functions' exponents, were less than one, which obviates calculation of $MRT$ as per Appendix Subsection Residence Time.

The GPC earliest, or start, time is $\beta$. The start times, $\beta$, of the PD (and thus of GPC distributions), known as the PD scale parameter could not be measured directly (no data at that time) and the unconstrained parameter introduced surfeit variability into the concentration and mass results. By testing $\beta$ every 5 s from 10 s to 35 s and plotting the errors of fit, a good compromise turned out to be an estimated circulatory first arrival time of drug of 25 to 30 s. The GPC harmonic mean residence time ($H\text{-}MRT$) was calculated as in Appendix Subsection Residence Time. Then $V_{H\text{-}MRT} = CL_{\text{GPC}}\, H\text{-}MRT$—see Table 2.

Equation (19) gave the statistical context of constant multipliers of the exponential terms of SET functions. For E2 models $c_1 e^{-\lambda_1 t} + c_2 e^{-\lambda_2 t} = AUC\left[p_1\left(\lambda_1 e^{-\lambda_1 t}\right) + p_2\left(\lambda_2 e^{-\lambda_2 t}\right)\right]$. In this, any $f(t) = \lambda e^{-\lambda t}$ term is a pdf, where $AUC_{\text{pdf}} = 1$. Then, $p_1 + p_2 = 1$, $p_1 = \frac{c_1\,\lambda_2}{c_1\,\lambda_2 + c_2\,\lambda_1}$ and $AUC_{C(t)} = \frac{c_2}{\lambda_2\, p_2}$. Biexponential fit parameters in this format appear in Table 3.

Table 3: Shown are biexponential (E2) statistical parameters from 7 dogs. Note that most of the $AUC$, a mean of 79.3%, is from the first term, and a lesser $AUC$ is from the second term, a mean of 20.7%.

| Case | $AUC$ | $p_1$ | $p_2$ | $\lambda_1$ | $\lambda_2$ |
|---|---|---|---|---|---|
| Units | $\frac{\text{mg}\cdot\text{h}}{\text{L}}$ | (%) | (%) | h$^{-1}$ | h$^{-1}$ |
| 1 | 18.6 | 82.3 | 17.7 | 0.725 | 0.0458 |
| 2 | 19.7 | 78.5 | 21.5 | 0.690 | 0.0402 |
| 3 | 8.8 | 81.6 | 18.4 | 0.742 | 0.0598 |
| 4 | 12.0 | 79.8 | 20.2 | 0.686 | 0.0457 |
| 5 | 16.0 | 73.9 | 26.1 | 0.886 | 0.0627 |
| 6 | 20.2 | 80.1 | 19.9 | 0.605 | 0.0427 |
| 7 | 15.2 | 78.5 | 21.5 | 0.714 | 0.0459 |
| mean | 15.8 | 79.3 | 20.7 | 0.721 | 0.0490 |
| SD | 4.2 | 2.7 | 2.7 | 0.085 | 0.0087 |

Figure 3: GPC fit relative errors (± is over/under estimate) plotted for sample-time groups in temporal sequence. Note the normal distribution of error, the narrow the 95% confidence intervals, and the closeness to zero error of the connected black circles of mean values for samples. The red connected circles are mean values of 5 left-out samples.

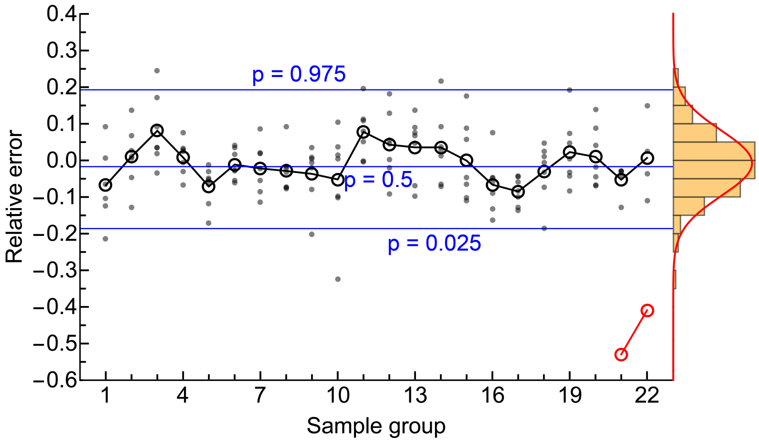


Figure 4: E2 fit relative errors (± is over/under estimate) plotted for sequential samples groups. Note the skewed distribution of error, the wide 95% confidence intervals, and the M-shaped variation of the connected black circles of mean values for samples. The mean values of 5 noisy samples left out not shown.

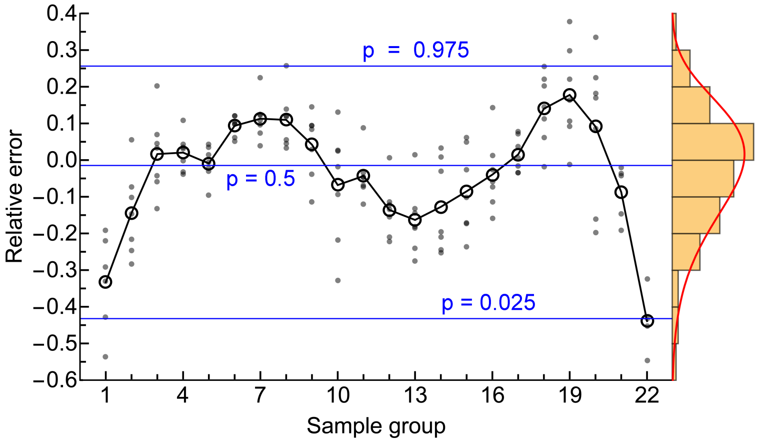


Fitting for proportional error was performed as per the Concentration data and fitting it Appendix Subsection. The results of the selection of the GPC and E2 fit functions and their parameters are next characterised by goodness of fit. As per Table 4, the GPC mean relative root mean square (rrms) error was 8.6%, where 10% or less was taken to be good *a priori* as elsewhere [37, 51], and where the residuals appear in Figure 3. The GPC residuals were narrow in range, and approximately normally distributed.

Biexponential model (E2) residual errors—see Figure 4—averaged 16.1% rrms or nearly twice that of the GPC fit error, using the same fitting methods. [3] The GPC intravenous bolus arrival time, $\beta$, was highly constrained, which adds only a fraction of a degree of freedom, *df*, to the 4 other parameters. To put it another way, using a 25 to 30 s window for $\beta$ gives an 8.61% residual error of fitting compared to 8.65% for a fixed 25 s time, which latter has exactly 4 *df* comparable to the 4 *df* of an E2, but which had a significantly smaller

[3]A display in sample groups may seem unusual, and the reader may be more familiar with residuals plotted over time. However, regression in $y$ alone generally injects correlation with $x$ when the $x$-values are not exactly equidistant, and to be seen, this has to be examined following display of residuals in the same way that regression sees them; as a sequence or list of values (that are then sum-squared and minimised).

rrms than E2's 16.1%. The E2 residuals are much more structured than the GPC residuals, see the Parameter errors from model-based bootstrap Appendix Section for details. The E2 model residuals had an overall 'M' shape, which was also identified for each individual E2 curve. The two 'M' humps are one each from the two local exponential density distributions of a biexponential (a triexponential has three humps). E2 model residuals were non-normally distributed (approximately a three parameter Weibull distribution) and had much wider 95% confidence interval than GPC residuals with an early and late time elongated tail in the underestimating direction (down in the figure). $AUC$ was thus systematically underestimated using E2 models. As implied by the structured residuals, SET models do not systematically approximate the derivatives of concentration. That is, biexponentials fit to this data had slopes roughly analogous to how a broken 24 h clock shows the correct time 3 times within 72 h—see Figure 5. This is the effect of not having explicit shape parameters in SET models; the shape of the data, i.e., the derivatives, are only transiently approximated.

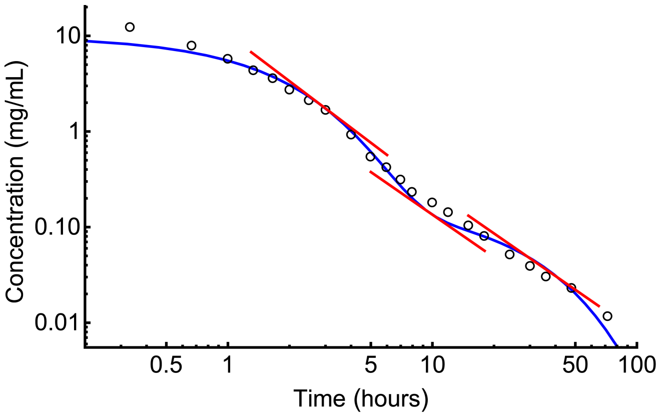


Figure 5: An example E2 fit to data (dog 6, blue curve). The black circles are the time samples. The approximate times for correct E2 slopes are shown at the point of tangency of the log-log tangent red line segments. Note the M-shaped wandering of the E2 function above and below the data locations, and especially the underestimation of sample concentration as well as incorrect slopes below the earliest and latest time samples.

Unexplained fraction is $1 - R^2$, a measure of goodness of fit, herein from the multiple correlations of $\ln(model)$ and $\ln(data)$—see Eq. (39). Table 4 shows these unexplained fractions from the E2 models (0.84% mean) to be multiple times greater than from GPC models (0.22% mean) for which the two-tailed Wilcoxon signed-rank of $p = 0.0156$ suggested significant difference.[4]

### Clearance

In addition to the fit errors, Table 4 shows clearance ($CL$) values for three models: the GPC, E2 and non-compartmental (NC) models, the latter as per the last paragraph of the Concentration data and fitting it Appendix Subsection. The NC $CL$ was significantly less

[4]Statistics: (1) A paired nonparametric test of a correlated sequence of $R^2$-values has greater power than comparing two $r$-values from independent random variables. (2) Without maximum likelihood fitting, and with different residual distributions an AIC (information criterion) comparison of E2 and GPC is intractable.

Table 4: Shown is clearance, $CL$ from biexponential, E2; noncompartmental, NC; and gamma-Pareto convolution, GPC, models. Relative root mean square (rrms) error of fit and unexplained fraction are shown as percentages for E2 and GPC.

| | $CL$ ($\text{ml}\cdot\text{min}^{-1}\cdot\text{kg}^{-1}$) | | | rrms error (%) | | unexplained fraction (%) | |
|---|---|---|---|---|---|---|---|
| Dog | E2 | NC | GPC | E2 | GPC | E2 | GPC |
| 1 | 16.4 | 12.3 | 9.8 | 20.4 | 8.7 | 1.24 | 0.17 |
| 2 | 16.5 | 15.2 | 11.5 | 15.9 | 6.3 | 0.78 | 0.10 |
| 3 | 37.0 | 30.6 | 26.7 | 11.8 | 5.9 | 0.47 | 0.11 |
| 4 | 27.1 | 22.7 | 19.4 | 14.0 | 13.8 | 0.65 | 0.50 |
| 5 | 20.3 | 17.5 | 12.4 | 10.6 | 9.5 | 0.45 | 0.29 |
| 6 | 16.1 | 14.4 | 11.4 | 16.7 | 6.1 | 0.76 | 0.09 |
| 7 | 21.3 | 18.4 | 14.7 | 23.1 | 10.0 | 1.57 | 0.29 |
| Mean | 22.1 | 18.7 | 15.1 | 16.1 | 8.6 | 0.84 | 0.22 |
| SD | 7.1 | 5.7 | 5.6 | 4.1 | 2.6 | 0.38 | 0.14 |

than the biexponential $CL$, but still significantly greater than the GPC $CL$ from Eq. (27) (both $p = 0.0078$, 1-tailed paired Wilcoxon tests). The NC and GPC correlation was good, $r^2 = 0.9840$, and the NC $CL$ was 3.6 $\text{ml}\cdot\text{min}^{-1}\cdot\text{kg}^{-1}$ greater than GPC $CL$. Note that for the GPC model $V_d'(t)$, i.e., rate of apparent volume growth with the same units as $CL$, achieved a limiting value of $6.0 \pm 0.6$ (mean $\pm$ SEM) times the $CL$.

### Drug mass

Table 5 shows GPC model relative $AUC$-values values at various times where 21.1% occurred

Table 5: $AUC$ for $C_{\text{GPC}}(t)$ curves as the percent of dose eliminated is shown for early time from 0 to 20 min, sample-times from 20 min to 72 h, late-time from 72 h to $\infty$, and from 0 to 72 h.

| Dog | $0 - \frac{1}{3}$ h | $\frac{1}{3} - 72$ h | 72 h$-\infty$ | $0 - 72$ h |
|---|---|---|---|---|
| 1 | 38.2 | 53.1 | 8.7 | 91.3 |
| 2 | 12.9 | 58.7 | 28.4 | 71.6 |
| 3 | 20.7 | 63.7 | 15.6 | 84.4 |
| 4 | 19.2 | 60.5 | 20.3 | 79.7 |
| 5 | 16.2 | 50.8 | 33.0 | 67.0 |
| 6 | 20.6 | 62.0 | 17.4 | 82.6 |
| 7 | 15.4 | 60.5 | 24.1 | 75.9 |
| Mean | 20.4 | 58.5 | 21.1 | 78.9 |
| SD | 8.3 | 4.8 | 8.2 | 8.2 |

following the last sample-time corresponding to 78.9% $\pm$ 8.2% elimination of the dose by 72 h. Figure 6 plots drug mass remaining from the GPC model Eq. (27) application of $M(t) = D\ S(t)$. Note, although dog 1 had the least $CL$ (9.8 $\text{ml}\cdot\text{min}^{-1}\cdot\text{kg}^{-1}$—Table 2) that dog had the greatest drug mass eliminated at 72 h (91.3%). Dog 1 *needs* less $CL$ to clear mass as that dog's volume of distribution from harmonic residence time, $V_{H\text{-}MRT}$, of 0.069 (L/kg)—Table 2—was an extremely reduced volume outlier. GPC's $V_{H\text{-}MRT}$ classifies volumes similarly to biexponential $V_{SS}$—see the Residence Time section of the Appendix for further information.

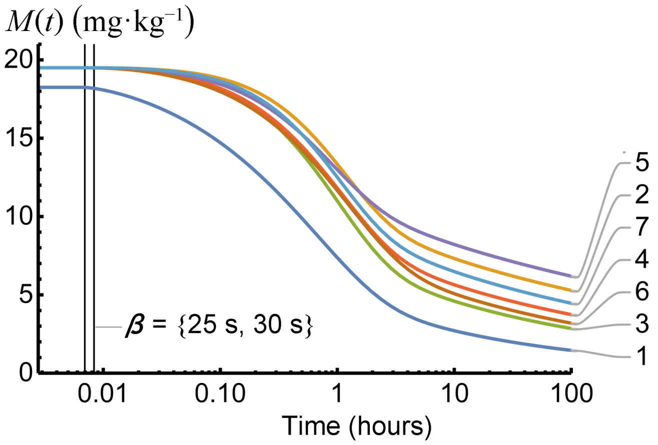


Figure 6: Linear-log plot of metformin (as base) mass retained in seven dogs as calculated from $D \cdot S(t)$ of the $\text{GPC}_{C(t)}$ model. Note how the dose (in mg per kg body weight) is retained in time. Note the constant mass until the Pareto density scale, $\beta$; the vascular first transit times of the models, occurred at 25 or 30 s. Dog 1 received a lesser dosage than the other dogs, and also rapidly cleared mass.

## Half-lives

Half-life defined at the instant in time it is measured was presented as Eq. (6) and in [23]. The fat-tailed GPC distribution, unlike lighter-tailed functions, yielded half-lives of mass and concentration that did not converge to the same value. Instead, they converged to different constants times the elapsed time—Eqs. (31) & (30)—where the ratio of those half-lives converged to a constant as shown in Figure 7. The time following peak ratio for 95% of the

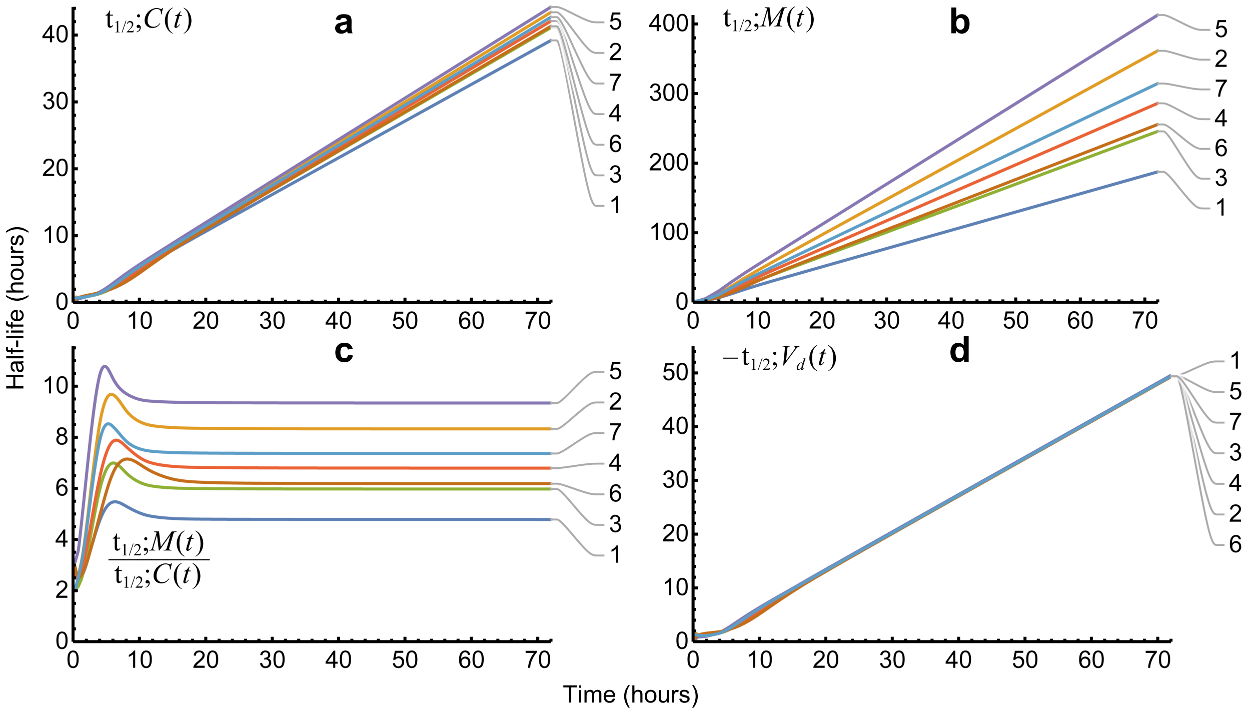


Figure 7: GPC model $t_{1/2}$ functions of time. Panel **a** shows $t_{1/2}; C(t)$; tightly grouped concentration $t_{1/2}$ functions. Panel **b** shows $t_{1/2}; M(t)$; more dispersed drug mass $t_{1/2}$ functions. Panel **c** shows the ratios of $t_{1/2}; M(t)$ to $t_{1/2}; C(t)$ converged to a mean of 7.0× by ≈ 10 h. Panel **d** shows that the (negative) half-life of $V_d(t)$ became proportional to $\ln 2\, t$, by ≈ 20 h.

ratio of half-lives of $M(t)$ and $C(t)$ to equal *terminal* occurred at a mean time of 9.8 h (range 7.4 to 13.1 h), well within the 72 h data collection time. The mean terminal ratio—*obtained without urine collection*—was 7.0 (range 4.8—9.3)—see Figure 7 and Eq. (9). The mean terminal ratio of mass to concentration half-lives is 1 for SET functions. Figure 8 shows this effect for our E2 models for which by 21 h 8 min the average $t_{1/2}; M(t)$ to $t_{1/2}; C(t)$ ratio collapsed to $< 1.001$ and rate of apparent volume growth, i.e., $V_d'(t)$, became vanishingly small as the models entered dynamic equilibrium. From Eq. (34) as illustrated in Figure 7d, the GPC models $-t_{1/2}; V_d(t)$ eventually converged to $\ln(2)\, t$. For an E2 model's $t_{1/2}; V_d(t)$ the equivalent asymptotes are

$$-t_{1/2}; V_d(t) \ \sim \ \ln(2) \frac{\lambda_2 p_2}{(\lambda_1 - \lambda_2)^2 p_1} \, e^{(\lambda_1 - \lambda_2)t}; \ \ t \to \infty \ ,$$

where $p_2 = 1 - p_1$ and where $\lambda_1 > \lambda_2$, which functions reached distinctly different asymptotes that grew much faster ($\approx 10^{19}$ by 72 h) than the single-valued-line GPC asymptote. Thus, the result for the GPC model that $-t_{1/2}; V_d(t) \sim \ln(2)\, t$ is simpler than the comparable results for $\mathrm{ED}_{n \geq 2}$ models.

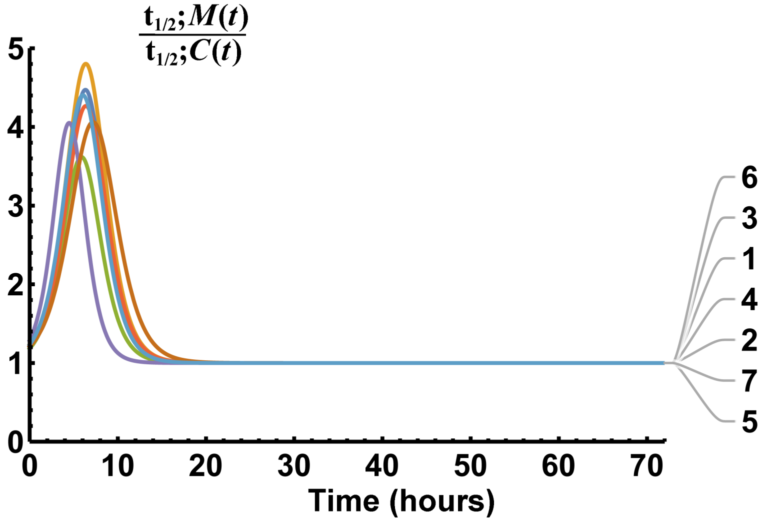


Figure 8: Shown are E2 mass and concentration half-life ratios for metformin in 7 dogs. Note the convergence of these ratios to 1 after approximately 21 hours. In effect, the two compartments function as a single compartment after that time and volume growth collapsed to trace amounts.

The Appendix Noncompartmental time-sample group, median half-life subsection provides the statistical properties of the noncompartmental, distribution-free method used to corroborate the trending of $t_{1/2}; C(t)$ values in time. (One does not use mean half-life values for this as they are unstable and *do not* measure centrality.) Figure 9 plots the stable noncompartmental (NC) median values at the geometric mean time for each pair of sample times of the each of 7 dogs' $t_{1/2}; C(t)$ for the earliest sample-time pair, then the next earliest and so forth for all 21 pairs of sample-time groups using Eq. (5). This plot is overlaid with the (stable) median values of $t_{1/2}; C_{\mathrm{GPC}}(t)$ model curves evaluated at those same geometric

Figure 9: This shows how the noncompartmental (NC) median half-lives of seven dogs vary with predicted median GPC model half-lives. The NC median $t_{1/2}; C(\mathbf{t})$ model values are shown as black circles. These are discrete serum concentration half-lives from adjacent time sample groups. Each pair of adjacent time sample-groups exhibited a different half-life with a definite overall trend for increased half-life for increasing time of measurement. More robust results were obtained from the continuous median GPC concentration half-life curve (blue) from all seven dogs.

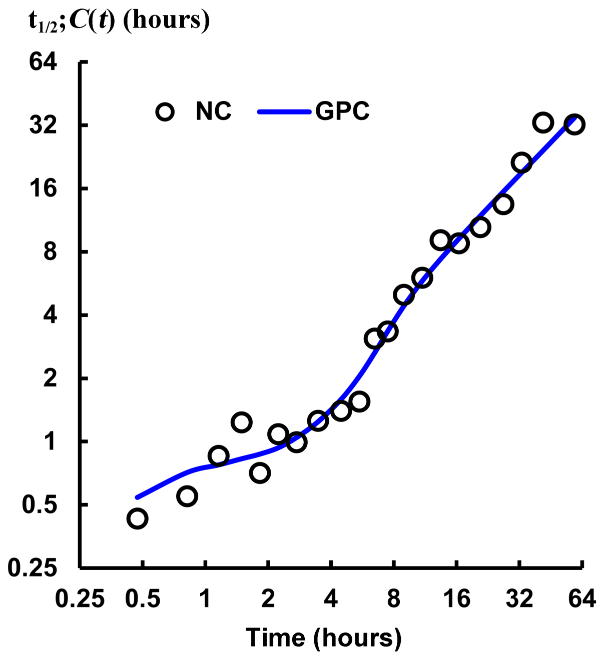


mean times. In that figure, the NC model median exhibited a significant trend for increasing values with elapsing time. There is independence between NC half-lives that do not share common time samples, i.e., the odd sample groups, $\{1, 3, 5, 7, ...21\}$, and the even sample groups $\{2, 4, 6, 8, ...22\}$ are each independent for trending. Thus, the overall trending of NC half-lives was from the data and not from any assumption made. That is, the NC model median $t_{1/2}; C(t)$ trending was due to metformin concentration kinetics. The GPC concentration half-lives smoothly followed the trending of the NC median $t_{1/2}; C(t)$ results, due to the two shape parameters in the GPC models that allowed reduced-error, derivative matching to serum concentration samples, which in turn allowed for accurate half-life functions to be calculated using Eq. (6). No convincing trend toward a terminal $t_{1/2}$-value was seen within the data. When expressed[5] parametrically $\ln\left[t_{1/2}; C_{\text{NC}}(t)\right] = 1.014\ \ln\left[t_{1/2}; C_{\text{GPC}}(t)\right]$ with 95% CI of 0.966 to 1.062 for slope, and a discarded, not significantly different from 0 intercept, had multiple correlation $R^2 = 0.9820$ and Pearson $r^2 = 0.9823$. Lin's concordance correlation [52] was $\rho_c = 0.9906$, i.e., in the almost perfect range of the McBride scale [53] of $\rho_c > 0.99$, with most of the $< 2\%$ error being NC noise. From this, these two very different measurements are almost perfectly measuring the same thing. To be clear, each adjacent sample group pair contained up to 14 samples for extraction of the NC median $t_{1/2}$. This has the *same* meaning as a plot of the terminal exponentials of NC models fit to data ending at the last time of that sample group pair, and, the more temporal data was included, the

[5]Logarithms were taken to reduce bias by creating approximately equal spacing of group-sample parametric values between those groups.

longer the $t_{1/2}$ was and Figure 9 shows that $t_{1/2}; C(t)$ *was* a function of time for our data that was also almost perfectly fit by the median GPC model. When this same NC data was parametrically log-log plotted with the median E2 $t_{1/2}; C(t)$, rather than median GPC models, the plot with NC $t_{1/2}; C(t)$ was a highly nonlinear sigmoidal function and the concordance dropped to $\rho_c = 0.9463$, or in the 0.90 to 0.95 moderate concordance range, and with no 95% confidence interval (CI) overlap with the GPC 95% $\rho_c$ CI's. That is, E2 $t_{1/2}; C(t)$ did not measure the same thing as $t_{1/2}; C(t)$ from GPC and/or sample group median NC $t_{1/2}$.

### Continuous Infusion Simulations

Constant infusion curves with limiting concentration, $C_{SS}$, were calculated indirectly from their IV bolus models' CDF as implied by Eq. (11). These are shown for the E2 and GPC models for dog 6 as Figure 10. For dog 6, the GCP infusion simulation of the right panel of Figure 10 suggests that metformin's persistent volume of distribution growth—see Eq. (4)—appeared to cause a major delay in the time ($t \gg 72$ h) it takes concentration to approach a steady state, $C_{SS}$, suggesting that measuring metformin $CL$ based on saturation during a constant infusion experiment would be intractable. However, constant infusion is considered by many to be a reference standard for measuring $CL$. To see why this might be the case, let us assume that the GPC model is the more accurate measurement and that we observe

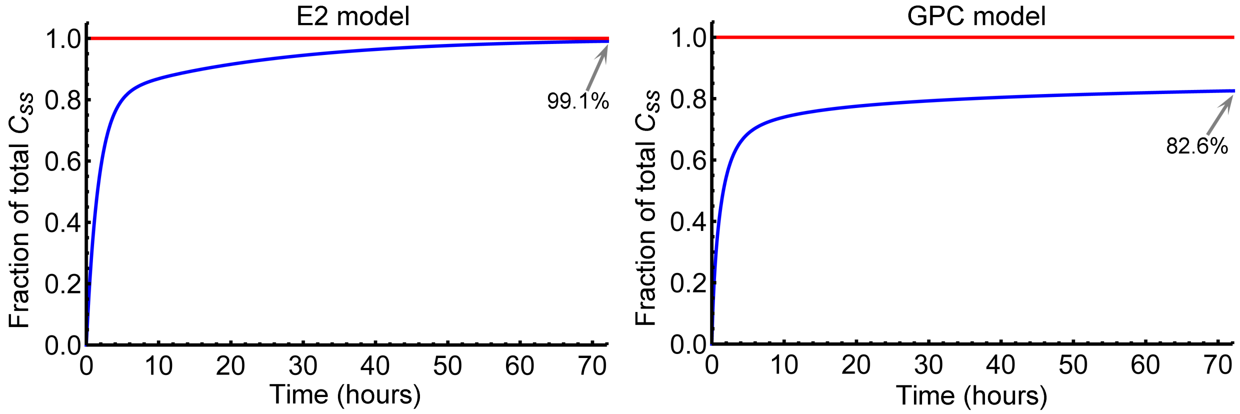


Figure 10: Shown are constant infusion simulations from a biexponential bolus model (**left panel**) a GPC bolus model (**right panel**) from dog 6 data. Each steady state concentration $C_{SS}$ is plotted as 100% (red lines). As seen in the figure, at 72 h the biexponential model is already at 99.1% of $C_{SS}$, whereas the GPC model is only at 82.6% at that same time.

a curve height of $C_{SS} = \dfrac{D_\mathrm{R}}{CL}$, Eq. (11). Thus, if one drew a reduced $C_{SS}$ height thinking that the data is from a quickly convergent E2 infusion function, a $CL$-value that is 20% too large ($0.991/0.826 - 1$) would be produced. From Table 4 the NC and E2 $CL$-values

were respectively 25% and 41% greater than the GPC $CL$-values for dog 6, such that a constant infusion measurement from an underestimated $C_{SS}$ would have appeared to reflect a better standard than other methods that also use exponential approximations to data. However, assigning an underestimated terminal asymptote can be more misleading in another context, i.e., the completeness of collection of drug mass in urine to establish the percent drug recovery. For that problem, estimating percent recovery from the administered dose is numerically much more reliable than from asymptotic completeness based on curve shape.

## Simulated dosing regimen for constant drug mass in the body

To illustrate a constant mean body drug burden during any dose interval, $\tau$, Eqs. (13) & (14) were applied to dog 6 data, which dog had moderate 72 h drug retention (17.4%—Table 5). The intravenous dose loading needed to maintain a constant average body drug mass in every $\tau = 12$ h dose interval is shown in Figure 11. As the plot is semi-log, a constant half-life would be a straight line, whereas in each dose interval, the plot is predominantly log-convex suggesting that the half-life increases for elapsed time during each dosing interval. As time progressed, the peak to trough ratios decreased and half-lives increased as per Eq. (6). Overall, the drug mass peak to trough variation varied less than one-tenth as much as it did for concentration for the same dosing intervals. The serial dose factor multipliers for constructing Figure 11 were calculated to be 4.478, 1.931, 1.172, 0.844, 0.668, 0.558, 0.482, 0.427, 0.385, 0.351. . . 0.136 times the body drug mass retained, where the latter number, 0.136, was the fractional dose administered under equilibrium conditions to maintain an average body burden of one dose during an eventual 12 h dose interval. This corresponds to an eventual $1/0.136 = 7.37$ times as much drug in the body as is being given in each dose, with the caveat that it would take 6 weeks of twice daily dog 6 dosing to drop to within 10% of terminal maintenance dosing.

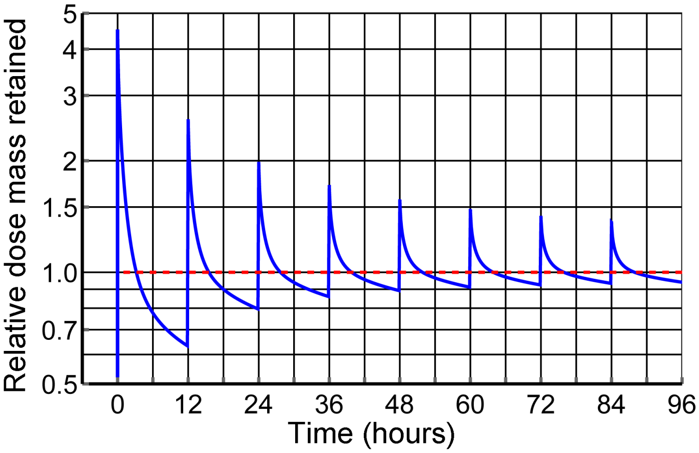

Figure 11: Semi-log plot of dog 6 simulations of Q12h IV metformin multidosing for unit mean dose mass in the body. The amplitude of peak-to-trough ratio decreased markedly with time from 7.1 during the first dose interval to 1.5 during the 84–96 h dose interval. See text for the constant mass loading dose regimen.

## Discussion

This work gathers together treatments of models of drug concentration as $AUC$ scaled density functions, variable apparent volumes of distribution equations with half-life defined as a real number variable of time. Also, the density function approach to convolution, a new definition for sums of exponential terms coefficients, an alternative expression for constant (inhomogeneous) volume of distribution from residence time that arises from a new density function model for concentration for power function tailed drugs; the gamma-Pareto type I convolution (GPC) were presented. The gamma-Pareto convolution (GPC) model chains a faster gamma distribution (GD) with a slower type I Pareto distribution (PD), the latter a normalised left truncated power function. As data during the first venous drug arrival times were not available, the $\beta$-parameter-values of the PD models were constrained to be 25—30 s and mimic reasonable vascular-drug arrival times. As per Table 2 both $a$ & $\alpha < 1$, and both the GD and PD were log-convex. However, their convolution; the GPC distributions, like the concentration curves they mimic, only became log-convex a short time after the model's peak concentration circa 2.7 min was achieved—see Table 2. The GD models were lighter-tailed than the first compartment E2 $\lambda_1$ but nonetheless augmented the GPC amplitudes out to relatively late times, circa 20 h—see Figure 1. GPC models are asymptotic with elapsing time to their second constituent distributions, the Pareto distribution (PD) power functions, Eq. (24). As $\alpha < 1$ for both the PD, and the GPC function asymptotic to it, their mean residence times ($MRT$) were undefined, and different mean times were found using harmonic $MRT$ ($H\text{-}MRT$). Unlike $V_d(t)$, which grew with time, $V_{H\text{-}MRT} = CL_{\text{GPC}}\, H\text{-}MRT$ were constant with values $< 1$ (L/kg) that were significantly Spearman rank correlated to E2 $V_{SS}$ values—see Table 2 and the Appendix Residence Time Subsection.

Neither non-equilibrated states nor variable volume modelling prohibit multicompartmental analysis. For example, a two-compartment GPC model would result from assigning a central compartment and a peripheral volume assigned that concentration as a function of time that conserves systemic mass. One might think that without classical multicompartmental modelling of cell membrane transport that pharmacogenomic effects would be undetectable. Dog 1 had the lowest $CL$ and highest 72 h mass eliminations while its terminal rate of volume growth, $V_d'(\infty)$, a parameter only meaningful for fat-tailed models, also had the least value in this short series of seven dogs. The reduced $V_d'(\infty)$-value indicates a relatively reduced rate of tissue absorption of the drug and may be due to a genetically deficient transporter protein. In effect, the requirement for conservation of mass in a single compartment variable volume model allowed physiological parsing into mass loss only to renal clearance and redistribution to be quantified as concentration dilution from apparent

volume growth. GPC distributions imply a single fractal recirculatory structure that could be elaborated mathematically. For an introduction to variable volume models and fractals the reader is urged read reference [54]. Although compartments and fractals are of theoretical interest, a model's *usefulness* arises from the scope of experimental results that it clarifies. Accordingly, this discussion focuses next on what GPC imply models, and how they might be used.

## Implications

The usual definition of renal clearance for a non-metabolised renal-only cleared marker is

$$CL_{\text{urine}} = \frac{\text{U V}}{\text{P}} \tag{35}$$

where U is drug concentration in urine, V is urine volume, and P is drug concentration in blood plasma. The assumption is that such a system is in dynamic equilibrium. U V/P for intravenous bolus conditions can be generalised to nonequilibrium states by multiplying it by either side of Eq. (9), i.e.,

$$CL = \frac{t_{1/2}; M(t)}{t_{1/2}; C(t)}\ \frac{\text{U V}}{\text{P}} = \frac{CL + V_d{}'(t)}{CL}\ \frac{\text{U V}}{\text{P}}\,. \tag{36}$$

To see this is so, one multiplies Eq. (10) by $t_{1/2}; C(t)$ to obtain the fractions of concentration that term-wise parse to mass elimination and volumetric dilution, as follows

$$1 = \overbrace{\frac{t_{1/2}; C(t)}{t_{1/2}; M(t)}}^{\text{Mass}} + \overbrace{\frac{t_{1/2}; C(t)}{-t_{1/2}; V_d(t)}}^{\text{Volume}} \tag{37a}$$

$$1 = \underbrace{\frac{CL}{CL + V_d'(t)}} + \underbrace{\frac{V_d'(t)}{CL + V_d'\,(t)}}\quad . \tag{37b}$$

Then, to correct plasma concentration, P, in the denominator of Eq. (35) to mass elimination conditions, we multiply its numerator by the reciprocals of the mass terms of Eqs. (37a) & (37b), *because these reciprocal mass terms are both sides* of Eq. (9), and are equal to each other. In other words, for a non-metabolised, renal-excreted drug, the molecules of drug that have shown up in urine, are only those molecules within the plasma that are parsed at that instant of time into urine as opposed to being 'redistributed' by ongoing apparent-volumetric concentration dilution.

How long the nonequilibrium state appears to persist depends on the model used. Monoexponential models lack nonequilibrium; they are instantly mixed. As all higher $\text{ED}_n$

models have an instantly mixed volume, a premature achievement of a terminal volume carries over to biexponential and higher models. In transient nonequilibrium $V_d'(t)$ eventually disappears—see Figure 8. To delay the time to dynamic equilibrium one can use NC models or heavier-tailed GD models. For example, a population biexponential model of a GFR marker was observed to have a 7.5 times shorter time to 95% of terminal mass half-life than GD models with enforced log-convexity [23].[6]

For a fat-tailed model, the nonequilibrium state is permanent and the ratio of half-lives achieved converged to $\frac{\alpha+1}{\alpha} = 7.0 \pm 1.5$ (mean $\pm$ standard deviation)—Eq. (32) & Table 2. As $\alpha$ (dimensionless) is the shape parameter of the Pareto distribution, and as the Pareto distribution is the GPC terminal tail asymptote, only the plasma concentration tail information would need to be extracted from data to quantify a late-time nonequilibrium $\frac{\mathrm{U\,V}}{\mathrm{P}}$ experiment. On average $CL = 7.0\,\frac{\mathrm{U\,V}}{\mathrm{P}}$ to within 5% for $t > 9.8$ h—see Figure 7c.

The other shape parameter from the gamma distribution (GD) part of the GPC model, $a$, aids in shape fitting of the early data. The two shape parameters of the GPC model allow for good derivative fitting between the data and the model; $C_{obs}(\mathbf{t})$ and $\mathrm{GPC}\left(\begin{smallmatrix} a & b \\ \alpha & \beta \end{smallmatrix} \,\middle|\, t\right)$. Even when GD models were used as a stand-alone; without convolution, provided that the $0 < a < 1$ shape had been selected by adaptive regularization, that shape parameter variation allowed matching to the different shapes of the disposition curves for fluid overloaded patients [55]. That E2 models were inefficient for metformin curve fitting was suggested by their poor goodness-of-fit—see Figures 3 & 4—and by their derivatives being unrelated to the data shape—see Figure 5. From Table 4, the E2 models averaged 16.1% rrms error with 0.84% unexplained fraction, the GPC models' errors were significantly better at 8.6% rrms with 0.22% unexplained fraction.

Thus far, there have been interesting implications from GPC modelling, for example, an apparent volume of distribution growth within the body that was terminally 6.0 times the renal clearance, that U V/P needed correction, that terminal half-life of mass appearance in urine was 7.0 times the serum drug concentration half-life value, that the drug mass retained in the body was substantial and that $V_d$ and $t_{1/2}$ increased with elapsed time during multidosing. Although direct testing of these findings was not within the scope of the current study, some of these results can be contrasted with prior study results, as follows.

[6] Adult human expected physical volume of drug distribution from mean residence time; E(V)=15-litres, clearance; $CL$=100-ml/min, using a glomerular filtration rate (GFR) marker; $^{169}$Yb-DTPA, from Table 1.

## Clearance

If metformin is only cleared by renal plasma flow (RPF) extraction, then its clearance should be ≤RPF. We estimated RPF from *p*-aminohippurate (PAH) dog data as PAH renal clearance divided by PAH arteriovenous extraction ratio, RPF $= \frac{CL_{\mathrm{PAH}}}{\mathrm{E_{PAH}}}$. Thus, $CL_{\mathrm{PAH}} = 13.51$-ml·min$^{-1}$·kg$^{-1}$ (mean, 75 dogs) [56] was divided by the $\mathrm{E_{PAH}} = 0.7564$ (metadata; weighted average 65 dogs) from three series [57–59] to yield RPF $\approx 17.86 \left(\frac{\mathrm{ml}}{\mathrm{kg \cdot min}}\right)$. From Table 4 the GPC model metformin $CL$ was 15.1 (ml·min$^{-1}$·kg$^{-1}$) or 84.8% of estimated RPF;[7] a result less than the 90%–100% metformin extraction fraction expected for humans [4]. There are multiple confounders, including possibly inaccurate $CL_{\mathrm{PAH}}$, and canine tubular resorption of PAH. However, the metformin extraction for the GPC model at 84.8% was greater than the nominal canine $\mathrm{E_{PAH}}$ of 75.6%, where the latter in humans is circa 90% and is called effective RPF. Less plausible and significantly greater results were seen for noncompartmental (NC) $CL$ of 18.7 ml·min$^{-1}$·kg$^{-1}$ at 104.7% of expected RPF with even worse results for E2 $CL$ at 22.1 (ml·min$^{-1}$·kg$^{-1}$) or 123.7% of expected RPF. We attribute the increased E2 $CL$-values to $AUC$ underestimation from under-extrapolation of concentration beyond the underestimated earliest and latest sample concentrations—see Figures 4 & 5.

The above results suggested that NC curve methods using exponential extrapolation largely prevented underestimation of slope magnitude under the time-samples themselves, but did not prevent a significant underestimation of $AUC$ in the extrapolated tails. This supported the proposition that heavier-tailed GPC models captured more of the otherwise under-extrapolated $AUC$—see Figure 3 and Table 5. From a prior work with a GFR marker, back-extrapolation using 120- to 240-min data validated with withheld early time-samples was more accurate using a $A - B\ln(t)$ fit function than using exponential back extrapolation, where the exponential back-extrapolation led to an underestimation of $AUC$ [36]. This agrees with the NC overestimation of $CL$-values seen elsewhere from exponential under-extrapolation of early $AUC$ [60] and of terminal tail areas [5, 25, 27]. Thus for multiple reasons, the $CL$-values from the GPC models seemed more plausible than the alternative models applied to the same data.

## Metformin drug mass

The survival of drug mass in the dogs' bodies at 72 h in Table 5 of 21.1% remaining represents a 5 fold decrease from the initial dose. By way of comparison, the serum concentration decreased 1000-fold from estimated peak concentration over 72 h—see Figure 1. As met-

[7] Geometric mean (GM) is a rarely-reported, better estimator of $CL$-values, and $\mathrm{GM}\left(\frac{\mathbf{CL}}{\mathbf{E}}\right) = \frac{\mathrm{GM}(\mathbf{CL})}{\mathrm{GM}(\mathbf{E})}$. Using GM $\mathrm{E_{PAH}}$ of 35 controls (of 65 above) yielded 84.7% (84.8% above) of RPF for GPC metformin.

formin extraction in the dog appeared to be less than estimated RPF, Clearance above, we assumed that drug mass decrease was from renal excretion. Thus, at 72 h on average 78.9% was GPC model renal excreted. Sheen and Sirtori et al. list human urinary metformin recoveries at 60 to 72 h, i.e., 86% and 78.9%—see Table 1 of [19] and Table II of [20]. A 99.9% 48 h urine recovery from IV metformin was calculated by Pentikäinen et al. [21], but may have been inflated from a graphic estimation of the location of maximum cumulative urine mass, thus subject to error as suggested in Figure 10 and surrounding text. Overall, the drug excretion implied here was similar to published human values, such that we cannot reject the hypothesis that the 72 h GPC model excreted metformin estimates were accurate.

## Metformin half-life

As summarised in Table 6 by collecting metformin in human urine Tucker et al. found 4.2 times longer mass than concentration half-life at 12 h [22] and Pentikäinen et al. [21] found a ratio of 5.1 times at 10 h. Collecting urine at 8 h Sirtori et al. found a ratio of 11.1 times [20]. During once a day multidosing of oral metformin in a PBPK model Robert et al. found an 8.8 times longer metformin half-life in erythrocytes, a mitochondrial depleted tissue marker, than in plasma [13]. In Table 6, the tissue (or urine) drug mass to plasma (or serum) ratios of half-lives averaged 7.3 ± 1.6 SEM. The GPC model ratios averaged 7.3 ± 0.1 SEM. The average E2 model half-life function ratios in this series at the same times $(8, 10, 12, 72\text{ h})$ would be $3.2, 1.9, 1.3, 1.0,$ which ratios are not as large as any of the published or GPC half-life ratios. Note that light-tailed modelling does not accommodate mass to concentration half-life ratios persistently greater than 1 until at least 168 h as RBC versus plasma $t_{1/2}$ in rats [4].

Table 6: Metformin $t_{1/2}$ in plasma [P, $C(t)$] and urine [U, $M(t)$] as $t_{1/2}; C(t)$ and ratios of $t_{1/2}$ of $C(t)$ and $M(t)$.

| Reference et al. | Species | Assay of | Last $t$ (h) | $t_{1/2}; C(t)$ (h) lit.* | $t_{1/2}; C(t)$ (h) GPC | $\frac{t_{1/2}; M(t)}{t_{1/2}; C(t)}$ lit. | $\frac{t_{1/2}; M(t)}{t_{1/2}; C(t)}$ GPC |
|---|---|---|---|---|---|---|---|
| Sirtori [20] | man | P, U | 8 | 1.52 | 3.70 | 11.1 | 7.6 |
| Pentikäinen [21] | man | P, U | 10 | 1.74 | 5.08 | 5.1 | 7.3 |
| Tucker [22] | man | P, U | 12 | 4.5 | 6.41 | 4.2 | 7.1 |
| Robert [13] † | man | P, RBC | 72 | — | — | 8.8 | — |
| Johnston [1] ‡ | dog | serum | 72 | 20.4 | 42.0 | — | 7.0 |

* lit. Literature value. GCP from this study only.
† Information extracted during once daily multidosing.
‡ Same concentration data used here.

The GPC model half-life of metformin serum concentration, Eq. (28), is asymptotically proportional to elapsed time, and predicts this trend with approximately twice the half-life observed for the exponential models in this series and in Table 6. This latter is not dissuasive as Figure 4 clearly shows biexponential underestimation of late time-samples,

which is consistent with underestimation of terminal half-lives for E2. Examination of the plot of median $t_{1/2}$ of grouped time-samples of metformin concentration half-lives in Figure 9 provided strong evidence that longer data collection times yielded longer half-lives. Table 6, Figures 9 & 8 show that $t_{1/2}$ of the mass and concentration, as first documented here, increased enough with elapsed time to provide a modelling agreement with experimentally observed ratios like $\dfrac{t_{1/2}; M(\infty)}{t_{1/2}; C(\infty)} = \dfrac{\alpha+1}{\alpha} > 1$ from persistent redistribution, $V_d'(\infty) = \dfrac{CL}{\alpha}$, i.e., old observations only now explained using new modelling concepts.

### A dosing strategy for possible use for therapy

In human 850 mg three times daily metformin for 5 days, in comparison to a single dose, Sambol et al. noted drug effect versus no (or lesser) effect for a single dose [24]. Also noted were the effects with elapsed time noted above, increased $C_{max}$, higher $V_d$ consistent with drug mass accumulation, and a prolongation of $t_{1/2}$ from 7.2 h to 19.8 h. The tentative explanation for this was 'The $V_d$...is probably larger after multiple doses...' Moreover, the lethargic pace adopted clinically of two weeks before changes are made to metformin dosing, the several months allotted to measuring outcomes in clinical trials [61], the relative sequestration of metformin into erythrocyte tissue versus plasma with elapsing time [13], the decreasing fasting plasma glucose for at least 8 weeks of multidosing [39] are consistent with the expanding apparent volume of drug distribution, increasing half-lives in time, and increasing sequestration of drug in tissue that form the basis for the Simulated dosing regimen for constant drug mass in the body subsection. Although diabetes, as present in Sambol et al.'s patients may reduce glucose transport and retard the time course of drug effects, another hypothesis would be that the majority drug effect is retarded due to slow tissue accumulation in all subjects, including our normal dogs. In any population, a faster initiation of therapeutic cytosol drug content may be available by using the dose loading method hypothesised in this paper, which may find eventual relevance for metformin dosing for cancer chemotherapy and/or other indications.

## Synopsis

Sum of exponential terms models have non-zero initial volumes problematic for calculating $t_{1/2}; M(t)$. For example, a monoexponential model has the same concentration half-life as its half-life of drug mass, $\dfrac{\ln(2)}{\lambda}$, and as $V_d'(t) = 0$, there is no volume growth (or redistribution). Using monoexponentials in 1959, Walser et al. [62] found a multiple day stubbornly persistent delay between plasma disappearance of radiolabeled urea and its appearance in urine of

approximately one hour. This implicates ongoing redistribution. It is only hindsight[8] that resolved the paradox in assuming no redistribution and finding irreducible redistribution. This adynamical constraint on apparent volume growth is only transiently mitigated by the inclusion of additional terms in a SET function, e.g., to 21 h for our E2, see Figure 8.

Herein, fat-tailed metformin models offered for the first time, results consistent with the observed half-life of mass appearance in urine as multiple times the serum drug concentration half-life values with drug mass versus serum drug concentration half-lives which agreed by their ratios and amounts to otherwise irreconcilable results. There were plausible drug masses at 72 h, a plausible explanation for increased $V_d$ and $t_{1/2}$ with multidosing with a plausible strategy for better multidosing for drug effects, and more plausible clearance-values than from the other methods tested. All of these things suggest potential usefulness for this type of modelling for extracting information and prediction that is problematic using current models for metformin kinetics.

We sought to determine why metformin half-lives did not appear to agree between publications, to develop a hypothesis concerning why metformin control of plasma glucose levels had delayed onset, e.g., following 4-weeks of oral dosing [16], and to speculate concerning the lack of a direct correlation between drug effect and blood metformin concentrations [17]. A single explanation for these phenomena may very well be that the mass half-life is persistently longer than the serum half-life and that mitochondria and cytosol are absorbing much of this mass, although reversibly. All these findings are consistent with high affinity of the drug in or in proximity to the sites of drug action.

## Limitations

The Introduction Section listed a number of references to drugs with heavy tails [5, 27–34, 63–66]. However, the potential clinical penetrance for fat-tailed data-spanning models is not known. For example, a power function tail for metformin does not seem to have been previously identified. There was no evidence for nonlinearity for the intravenous route of administration at the drug levels in this study. However, nonlinearity might occur at higher drug levels. A PBPK-type study where blood, urine, and tissues are all collected in an animal study may be appropriate for further investigation. Only a prospective study that measures both drug mass and drug effect can be used to validate how useful the drug mass with drug effect relationship hypothesised for the GPC model is for metformin.

The assay used allowed for 72 hours of serum data collection [67]. Due to a technical error, the earliest time-samples were at 20 min [1]. Approximately 20.4% of the $AUC$ occurred

[8]Walser et al.'s excellent work related the delay between plasma disappearance of urea and its appearance in urine as being unexplained.

before 20-min—see Table 5. This was verified indirectly by comparing the $CL$ to renal plasma flow as estimated from metadata, implying that a lot of this early $AUC$ would be missed using NC methods. Due to this early time back-extrapolation some parameters may be somewhat imprecise but not others, e.g., $AUC_0^{20\text{-min}}$ but not $t_{1/2}; C(t > 20\text{-min})$. The last two time-samples were erratic especially for dogs 3, 4 and 5 but not for dogs 1, 2, 6 and 7 as seen in Figure 1. The apparent increased magnitude and variability of some of the last two time-samples had no clear cause and was not prior identified [1].

The GPC model with simplex method global search optimisation has a long run time, and further refinement of the algorithm for speed would be useful. As a new PK model convergent to a power function tail, the GPC model has only been applied to metformin. How well the GPC model would estimate the plasma/serum concentrations of other intravenously injected substances is unknown at this time. In common with other first applications of a fit function for intravenous drug dosing, there was no modelling of oral dose data, tissue drug concentrations, or correlation with simultaneously measured drug excreted in urine, the applicability is only for linear systems, population kinetics were not explored in the usual sense, and the fitting algorithm used does not allow for case-wise error statistics of parameters, *per se*, necessitating calculation by other means, for example, as appears in the Parameter errors from model-based bootstrap Appendix Section.

## Conclusion

Fat-tailed models of metformin concentration were fit to data from 7 dogs. The fat tails altered the usual context of some pharmacokinetic parameters. Metformin was cleared at 84.8% of estimated total renal plasma flow. At 72 h there was 21.1% unexcreted metformin. Half-lives eventually became linear functions of the sample-times. The mean ratio of mass to concentration $t_{1/2}$ 95% converged to 7.0 by 9.8 h. Multidosing for constant drug mass rather than serum drug levels was simulated and may allow for an earlier onset and better maintenance of drug effects but is in need of validation.

## Acknowledgements

The authors thank Charlotte A. Johnston, DVM; Valerie S. MacDonald Dickinson, DVM and M. Casey Gaunt, DVM as well as the American Journal of Veterinary Research for permission to use data collected, analysed and published in reference [1].

# References


1. Johnston CA, Dickinson VSM, Alcorn J, Gaunt MC (2017) Pharmacokinetics and oral bioavailability of metformin hydrochloride in healthy mixed-breed dogs. Am J Vet Res 78(10):1193–1199
2. Wypij JM (2017) Pilot study of oral metformin in cancer-bearing cats. Veterinary and Comparative Oncology 15(2):345–354
3. Andrzejewski S, Gravel SP, Pollak M, St-Pierre J (2014) Metformin directly acts on mitochondria to alter cellular bioenergetics. Cancer & Metabolism 2(1):12
4. Xie F, Ke AB, Bowers GD, Zamek-Gliszczynski MJ (2015) Metformin's intrinsic blood-to-plasma partition ratio (B/P): reconciling the perceived high in vivo B/P> 10 with the in vitro equilibrium value of unity. J Pharmacol Exp Ther 354(2):225–229
5. Kajbaf F, Bennis Y, Hurtel-Lemaire AS, Andréjak M, Lalau JD (2016) Unexpectedly long half-life of metformin elimination in cases of metformin accumulation. Diabet Med 33(1):105–110 http://dx.doi.org/10.1111/dme.12959
6. Wang DS, Kusuhara H, Kato Y, Jonker JW, Schinkel AH, Sugiyama Y (2003) Involvement of organic cation transporter 1 in the lactic acidosis caused by metformin. Molecular Pharmacology 63(4):844–848 http://molpharm.aspetjournals.org/content/63/4/844
7. Kimura N, Okuda M, Inui Ki (2005) Metformin transport by renal basolateral organic cation transporter hOCT2. Pharmaceutical Research 22(2):255–259
8. MacKenzie MJ, Ernst S, Johnson C, Winquist E (2012) A phase I study of temsirolimus and metformin in advanced solid tumours. Invest New Drugs 30(2):647–652
9. He L, Wondisford FE (2015) Metformin action: concentrations matter. Cell Metab 21(2):159–162
10. Bailey CJ, Wilcock C, Scarpello JHB (2008) Metformin and the intestine. Diabetologia 51(8):1552
11. Graham GG, Punt J, Arora M, Day RO, Doogue MP, Duong J, Furlong TJ, Greenfield JR, et al. (2011) Clinical pharmacokinetics of metformin. Clin Pharmacokinet 50(2):81–98 http://dx.doi.org/10.2165/11534750-000000000-00000
12. Moreira PI (2014) Metformin in the diabetic brain: friend or foe? Annals of Translational Medicine 2(6)
13. Robert F, Fendri S, Hary L, Lacroix C, Andrejak M, Lalau JD (2003) Kinetics of plasma and erythrocyte metformin after acute administration in healthy subjects. Diabetes & Metabolism 29(3):279–283
14. Madiraju AK, Erion DM, Rahimi Y, Zhang XM, Braddock D, Albright RA, Prigaro BJ, Wood JL, et al. (2014) Metformin suppresses gluconeogenesis by inhibiting mitochondrial glycerophosphate dehydrogenase. Nature 510(7506):542
15. Owen MR, Doran E, Halestrap AP (2000) Evidence that metformin exerts its anti-diabetic effects through inhibition of complex 1 of the mitochondrial respiratory chain. Biochem J 348(3):607–614

16. Buse JB, DeFronzo RA, Rosenstock J, Kim T, Burns C, Skare S, Baron A, Fineman M (2016) The primary glucose-lowering effect of metformin resides in the gut, not the circulation: results from short-term pharmacokinetic and 12-week dose-ranging studies. Diabetes Care 39(2):198–205
17. Stepensky D, Friedman M, Raz I, Hoffman A (2002) Pharmacokinetic-pharmacodynamic analysis of the glucose-lowering effect of metformin in diabetic rats reveals first-pass pharmacodynamic effect. Drug Metab Dispos 30(8):861–868
18. Lodish H, Berk A, Zipursky SL, Matsudaira P, Baltimore D, Darnell J (2003). Section 5.3, Organelles of the eukaryotic cell In: Lodish H, (ed). Molecular cell biology, 5th edition. Freeman WH, p. 171 https://www.ncbi.nlm.nih.gov/books/NBK21743/
19. Scheen AJ (1996) Clinical pharmacokinetics of metformin. Clin Pharmacokinet 30(5):359–371
20. Sirtori CR, Franceschini G, Galli-Kienle M, Cighetti G, Galli G, Bondioli A, Conti F (1978) Disposition of metformin (N,N-dimethylbiguanide) in man. Clinical Pharmacology & Therapeutics 24(6):683–693 http://dx.doi.org/10.1002/cpt1978246683
21. Pentikäinen PJ, Neuvonen PJ, Penttilä A (1979) Pharmacokinetics of metformin after intravenous and oral administration to man. Eur J Clin Pharmacol 16(3):195–202 http://dx.doi.org/10.1007/BF00562061
22. Tucker GT, Casey C, Phillips PJ, Connor H, Ward JD, Woods HF (1981) Metformin kinetics in healthy subjects and in patients with diabetes mellitus. Br J Clin Pharmacol 12(2):235–246
23. Wesolowski CA, Wesolowski MJ, Babyn PS, Wanasundara SN (2016) Time varying apparent volume of distribution and drug half-lives following intravenous bolus injections. PLoS ONE 11(7):e0158798 http://dx.doi.org/10.1371%2Fjournal.pone.0158798
24. Sambol NC, Chiang J, O'conner M, Liu CY, Lin ET, Goodman AM, Benet LZ, Karam JH (1996) Pharmacokinetics and pharmacodynamics of metformin in healthy subjects and patients with noninsulin-dependent diabetes mellitus. The Journal of Clinical Pharmacology 36(11):1012–1021
25. Wesolowski CA, Puetter RC, Ling L, Babyn PS (2010) Tikhonov adaptively regularized gamma variate fitting to assess plasma clearance of inert renal markers. J Pharmacokinet Pharmacodyn 37(5):435–474
26. Wesolowski CA, Babyn PS, Puetter RC, inventors; Carl A. Wesolowski, assignee (2014) Method for evaluating renal function. US Patent 8,738,345
27. Ghobadi C, Mirhosseini N, Shiran MR, Moghadamnia A, Lennard MS, Ledger WL, Rostami-Hodjegan A (2009) Single dose pharmacokinetic study of clomiphene citrate isomers in anovular patients with polycystic ovary disease. The Journal of Clinical Pharmacology 49(2):147–154 http://dx.doi.org/10.1177/0091270008328096
28. Anderson J, Tomlinson RWS (1966) The distribution of calcium-47 in the rat. The Journal of Physiology 182(3):664–670 http://www.ncbi.nlm.nih.gov/pmc/articles/PMC1357493/
29. Anderson J, Osborn SB, Tomlinson RWS, Wise ME (1969) Clearance curves for radioactive

tracers - sums of exponentials or powers of time? Physics in Medicine & Biology 14(3):498 http://stacks.iop.org/0031-9155/14/i=3/a=415

30. Norwich KH, Siu S (1982) Power functions in physiology and pharmacology. J Theor Biol 95(2):387–398 http://www.sciencedirect.com/science/article/pii/0022519382902533
31. Wise ME (1985) Negative power functions of time in pharmacokinetics and their implications. J Pharmacokinet Biopharm 13(3):309–346 http://dx.doi.org/10.1007/BF01065658
32. Norwich KH (1997) Noncompartmental models of whole-body clearance of tracers: A review. Ann Biomed Eng 25(3):421–439 http://dx.doi.org/10.1007/BF02684184
33. Dokoumetzidis A, Magin R, Macheras P (2010) Fractional kinetics in multi-compartmental systems. J Pharmacokinet Pharmacodyn 37(5):507–524 http://dx.doi.org/10.1007/s10928-010-9170-4
34. Dokoumetzidis A, Macheras P (2011) The changing face of the rate concept in biopharmaceutical sciences: from classical to fractal and finally to fractional. Pharmeceutical Research 28(5):1229–1232 http://dx.doi.org/10.1007/s11095-011-0370-4
35. Macheras P (1995) Carrier-mediated transport can obey fractal kinetics Pharmaceutical Research 12(4):541–548
36. Wanasundara SN, Wesolowski MJ, Puetter RC, Burniston MT, Xirouchakis E, Giamalis IG, Babyn PS, Wesolowski CA (2015) The early plasma concentration of $^{51}$Cr-EDTA in patients with cirrhosis and ascites: a comparison of three models. Nucl Med Commun 36(4):392
37. Wesolowski CA, Wanasundara SN, Wesolowski MJ, Erbas B, Babyn PS (2016) A gamma-distribution convolution model of $^{99m}$Tc-MIBI thyroid time-activity curves. EJNMMI Physics 3(1):31 https://www.ncbi.nlm.nih.gov/pmc/articles/PMC5161052/
38. Sopasakis P, Sarimveis H, Macheras P, Dokoumetzidis A (2018) Fractional calculus in pharmacokinetics Journal of pharmacokinetics and pharmacodynamics 45(1):107–125
39. McGill JB, Cole TG, Nowatzke W, Houghton S, Ammirati EB, Gautille T, Sarno MJ (2004) Circulating 1, 5-anhydroglucitol levels in adult patients with diabetes reflect longitudinal changes of glycemia: a US trial of the GlycoMark assay. Diabetes Care 27(8):1859–1865
40. Niazi S (1976) Volume of distribution as a function of time. J Pharm Sci 65(3):452–454
41. Wesolowski CA, Hogendoorn P, Vandierendonck R, Driedger AA (1988) Bolus injections of measured amounts of radioactivity. Journal of Nuclear Medicine Technology 16(1):1–4
42. Horner J, Lykawka PS (2010) 2001 QR322: a dynamically unstable Neptune Trojan? Mon Not R Astron Soc 405(1):49–56
43. Chelouah R, Siarry P (2003) Genetic and Nelder–Mead algorithms hybridized for a more accurate global optimization of continuous multiminima functions European Journal of operational research 148(2):335–348
44. Orlando R, Floreani M, Padrini R, Palatini P (1998) Determination of inulin clearance by bolus intravenous injection in healthy subjects and ascitic patients: equivalence of systemic and renal clearances as glomerular filtration markers. Br J Clin Pharmacol 46(6):605

45. Florijn KW, Barendregt JNM, Lentjes EGWM, van Dam W, Prodjosudjadi W, van Saase JLCM, van Es LA, Chang PC (1994) Glomerular filtration rate measurement by "single-shot" injection of inulin. Kidney Int 46(1):252–259
46. Toutain PL, Bousquet-Mélou A (2004) Plasma terminal half-life. J Vet Pharmacol Ther 27(6):427–439 http://dx.doi.org/10.1111/j.1365-2885.2004.00600.x
47. Wanasundara SN, Wesolowski MJ, Barnfield MC, Waller ML, Murray AW, Burniston MT, Babyn PS, Wesolowski CA (2016) Accurate and precise plasma clearance measurement using four $^{99m}$Tc-DTPA plasma samples over 4 h. Nucl Med Commun 37(1):79
48. Nadarajah S, Kotz S (2007) On the convolution of Pareto and gamma distributions Computer Networks 51(12):3650–3654
49. Wells J (2013). Of fat cats and fat tails: From the financial crisis to the 'new' probabilistic Marxism In: Zarembka P, (ed). Contradictions: finance, greed, and labor unequally paid. Emerald Group Publishing Limited p. 197–228
50. Vander Zanden JW (1960) Pareto and fascism reconsidered. American Journal of Economics and Sociology 19:399–411
51. Burger D, Ewings F, Kabamba D, L'homme R, Mulenga V, Kankasa C, Thomason M, Gibb DM, et al. (2010) Limited sampling models to predict the pharmacokinetics of nevirapine, stavudine, and lamivudine in HIV-infected children treated with pediatric fixed-dose combination tablets Therapeutic drug monitoring 32(3):369–372
52. Lawrence I, Lin K (1992) Assay validation using the concordance correlation coefficient. Biometrics p. 599–604
53. McBride GB (2005) A proposal for strength-of-agreement criteria for Lin's concordance correlation coefficient. NIWA Client Report: HAM2005-062, National Institute of Water & Atmospheric Research Ltd, Hamilton, New Zealand
54. Macheras P, Iliadis A (2016). Chapter 7: Emperical Models In: Macheras P, Iliadis A, (eds). Modeling in Biopharmaceutics, Pharmacokinetics and Pharmacodynamics Springer p. 161–189
55. Wesolowski CA, Ling L, Xirouchakis E, Burniston MT, Puetter RC, Babyn PS, Giamalis IG, Burroughs AK (2011) Validation of Tikhonov adaptively regularized gamma variate fitting with 24-h plasma clearance in cirrhotic patients with ascites. Eur J Nucl Med Mol Imaging 38(12):2247–2256
56. Houck CR (1948) Statistical analysis of filtration rate and effective renal plasma flow related to weight and surface area in dogs. American Journal of Physiology–Legacy Content 153(1):169–175
57. Moghissi K, Machell ES, Munday KA (1969) Changes in renal blood flow and PAH extraction during extracorporeal circulation of short and long duration. Cardiovasc Res 3(1):37–44
58. Earley LE, Friedler RM (1965) Changes in renal blood flow and possibly the intrarenal distribution of blood during the natriuresis accompanying saline loading in the dog. The Journal of Clinical Investigation 44(6):929–941

59. Slick GL, Aguilera AJ, Zambraski EJ, DiBona GF, Kaloyanides GJ (1975) Renal neuroadrenergic transmission. American Journal of Physiology-Legacy Content 229(1):60–65
60. Wickham F, Burniston MT, Xirouchakis E, Theocharidou E, Wesolowski CA, Hilson AJW, Burroughs AK (2013) Development of a modified sampling and calculation method for isotope plasma clearance assessment of the glomerular filtration rate in patients with cirrhosis and ascites. Nucl Med Commun 34(11):1124–1132
61. U S Food and Drug Administration and others (2008) Glucophage (metformin hydrochloride tablets)/Glucophage XR (metformin hydrochloride extended release tablets). NDA 20-357/S-031 and NDA 21-202/S-016. Bristol-Myers Squibb, Princeton (NJ) p. 3–32
62. Walser M, Bodenlos LJ (1959) Urea metabolism in man. The Journal of Clinical Investigation 38(9):1617–1626
63. Claret L, Iliadis A, Macheras P (2001) A stochastic model describes the heterogeneous pharmacokinetics of cyclosporin. Journal of Pharmacokinetics and Pharmacodynamics 28(5):445–463 http://dx.doi.org/10.1023/A:1012295014352
64. Fuite J, Marsh R, Tuszyński J (2002) Fractal pharmacokinetics of the drug mibefradil in the liver. Physical Review E 66(2):021904 http://link.aps.org/doi/10.1103/PhysRevE.66.021904
65. Popović JK, Atanacković MT, Pilipović AS, Rapaić MR, Pilipović S, Atanacković TM (2010) A new approach to the compartmental analysis in pharmacokinetics: fractional time evolution of diclofenac. J Pharmacokinet Pharmacodyn 37(2):119–134 http://dx.doi.org/10.1007/s10928-009-9147-3
66. Weiss M (1999) The anomalous pharmacokinetics of amiodarone explained by nonexponential tissue trapping. Journal of Pharmacokinetics and Biopharmaceutics 27(4):383–396 http://dx.doi.org/10.1023/A:1020965005254
67. Michel D, Gaunt MC, Arnason T, El-Aneed A (2015) Development and validation of fast and simple flow injection analysis–tandem mass spectrometry (FIA–MS/MS) for the determination of metformin in dog serum. J Pharm Biomed Anal 107:229–235
68. Veng-Pedersen P (1977) Curve fitting and modeling in pharmacokinetics and some practical experiences with NONLIN and a new program FUNFIT. J Pharmacokinet Biopharm 5(5):513–531
69. Downey AB Evidence for long-tailed distributions in the internet In: Proceedings of the 1st ACM SIGCOMM Workshop on Internet Measurement ACM (2001). p. 229–241
70. Mantel N (1976) Tails of distributions The American Statistician 30(1):14–17
71. Mooney CZ (1996) Bootstrap statistical inference: Examples and evaluations for political science American Journal of Political Science 40(2):570
72. Green R, Hahn W, Rocke D (1987) Standard errors for elasticities: a comparison of bootstrap and asymptotic standard errors Journal of Business & Economic Statistics 5(1):145–149

# Appendix

## Concentration data and fitting it

The samples ranged from 9.78–19100 ng/ml metformin. The 20 min sample for dog three was discarded due to quality assurance failure in the original study. In the current study, probable quality assurance failure was additionally identified with squared rank testing in five of 14 of the last two sample-time groups (at 48 and 72 h) leading to discard. Thus, a total of 148 samples of the original 154 (96%) were used for analysis here. Serum samples were analysed using a simple flow injection analysis–tandem mass spectrometry (FIA–MS/MS) by Michel et al. [67], also developed at the University of Saskatchewan. This method has linear response from lowest limit of quantification (LLOQ) of 5 ng/ml to a maximum of 2340 ng/ml metformin base with a relative root mean square of proportional error (rrms) of 5.2% calibration proportional error. The dog doses were weighed and diluted as metformin hydrochloride (165.625 DA average molecular weight) and dose mass corrected to metformin base (129.164 DA average molecular weight) for the computations in this publication.

Noncompartmental analysis of $AUC$ and $CL$ was performed with Prism 5.0 (GraphPad Prism, San Diego, CA, USA) for Johnston et al. [1] and reused here. The $AUC$ values were from the log-trapezoidal rule, with 4-10 early and late time-sample (a generous number) used for extrapolation. This NC technique has the advantage that it is commonly used, but should not be considered optimally comparable to proportional modelling used with better fitting and extrapolating functions than monoexponentials.

For GPC and E2 modelling, multiple regression weightings were tested including ordinary least squares (OLS), Poisson weighting—see [37], OLS fitting of the logarithms of concentration and finally proportional weighting that reduces the relative error and produced the best fitted results. Thus, the GPC and E2 models were fitted using the minimum $\text{L}_2$ norm of relative error of residuals. That is, for the vector $C_{\text{obs}}(\mathbf{t})$, we sought $C(\cdots;\mathbf{t}) = \kappa\ \text{pdf}(\mathbf{t};\cdots)$ using

$$\min \left\| \frac{C(\cdots;\mathbf{t})}{C_{obs}(\mathbf{t})} - 1 \right\|_2 \quad . \tag{38}$$

The $\text{L}_2$ norm is a monotone transform (square root) of the least squares norm, $\text{L}_2^2$. Formula (38) finds the $1/C_{\text{obs}}^2(\mathbf{t})$ weighted least squares solutions used elsewhere [37, 44–47, 68]. The error metric for formula (38) was the root mean square of proportional error (rrms),

$$\text{rrms\%} = 100 \min \sqrt{\sum_{i=1}^{n} \frac{1}{n} \left[ \frac{f(x_i) - y_i}{y_i} \right]^2} = \frac{100}{\sqrt{n}} \min \left\| \frac{f(\mathbf{x})}{\mathbf{y}} - 1 \right\|_2 ,$$

where the right hand equation converts between the absolute value form with $y_i^2$ in the denominator (whence the $1/C_{obs}^{\,2}(\mathbf{t})$ notation) to $L_2$ vector notation format. Both the metformin assay (5.2% rrms) [67], and the GPC residuals (8.6% rrms)—see Table 4 and Figure 3—exhibited proportional error.[9] The $L_2$ proportional norm is related its logarithmic norm as follows. Let $\frac{f(\mathbf{x})}{\mathbf{y}} = u$. Expanding $\ln(u)$ around $u = 1$ for the $i^{\text{th}}$ term being odd yields

$$\ln(u) = (u-1) - \frac{1}{2}(u-1)^2 + \frac{1}{3}(u-1)^3 - \cdots + \frac{1}{i}(u-1)^i - \ldots; \;\; 0 < u \leq 2\,. \tag{39}$$

This expansion $\ln\frac{f(\mathbf{x})}{\mathbf{y}} \approx (u-1) = \frac{f(\mathbf{x})}{\mathbf{y}} - 1$, has as its first term the target for the proportional norm minimisation of relative error. Proportional error values closer to 0 are asymptotically $\ln\frac{f(\mathbf{x})}{\mathbf{y}} = \ln f(\mathbf{x}) - \ln(\mathbf{y})$. Thus, log transformations of $f(\mathbf{x})$ and $\mathbf{y}$ were used to calculate $1 - R^2$ values to make unexplained fractions of total variance indexed to goodness-of-fit.

## Relative tail heaviness

The limiting ratio as elapsed time increases of two competing models' survival functions is the statistic of choice for testing relative tail heaviness applicable to density functions, mass functions, functions with discontinuities, functions without derivatives, piecewise defined distributions, and empirical distributions. The use of classification schemes, i.e., indirect comparisons using derivatives and relying upon hazard function theorems, is an unreliable substitute for the use of smoother integral methods for random variable data using survival functions [69]. For continuous functions, pdf binary comparison through survival function ratios avoids false attribution, for example, classifying the GD as having an ED terminal tail, whereas in fact, the exponential has tail heaviness that is within the GD tail heaviness spectrum.[10] This is shown theoretically as follows.

The relative terminal tail heaviness of the $S_{\text{GD}} = Q(a, b\,t)$, Eq. (16), and the monoexponential survival function, $S_{\text{ED}} = e^{-\lambda\,t}$, is obtained from their ratio

$$\frac{S_{\text{GD}}(t)}{S_{\text{ED}}(t)} = \frac{Q(a, b\,t)}{e^{-\lambda\,t}} \;\;, \tag{40}$$

which is a $\frac{0}{0}$ type indeterminate form, for $t \to \infty$. Applying L'Hôpital's rule yields

$$\frac{b}{\lambda\,\Gamma(a)}\frac{e^{(\lambda-b)\,t}}{(b\,t)^{1-a}} \;\;. \tag{41}$$

[9] OLS regression failed Veng Pedersen's sign run criterion for the terminal tail [68] confirming Toutain and Bousquet-Mélou's (Figure 17) [46] recommendation to fit data for relative error.

[10] Some authors, for $\lim_{t\to\infty}\frac{f(t)}{S(t)}$, where $f(t)$ is a density function and $S(t)$ is its survival function, mistakenly apply L'Hôpital's rule to that limit without having an indeterminate form. The confusion likely arises from the ED's unique property; $\frac{f(t)}{S(t)} = \frac{f'(t)}{S'(t)} = \lambda$, whereas in general $\frac{f(t)}{S(t)} \neq \frac{f'(t)}{S'(t)}$.

For $a, b, \lambda > 0$, the limit as $t \to \infty$ of the right hand fraction of this is dominated by the exponential, which goes to infinity for $\lambda > b$ and to zero when $\lambda < b$, which means that the GD tail is only heavier than the ED tail when $\lambda > b$. $\lambda \equiv \beta$ does not occur following regression because as $\beta \to \lambda$, then $\alpha \to 1$, and GD becomes statistically indistinguishable from ED. Thus, ED has a GD spectrum tail heaviness, not the obverse.

Experimental demonstration of this theoretical result includes the following. $\lambda > b$ always occurred only when special methods were used for GD fitting. The slower E2 rate constant, $\lambda_2 > b$, with $b$ from adaptively regularised GD fitting that minimised $AUC$ error; mean $\lambda_2 = 0.275 > b = 0.216$ h$^{-1}$ [23]. Herein, the GD tails were lighter even than the E2 fast decays, i.e., all $\lambda_1 < b$.

Classifications of tail heaviness consist of broad categories of very different functions each having a different spectrum of tail heaviness. Even so, the exponential distribution tail heaviness category did not overlap with the category for Pareto distributions [70]. Even when two functions are in the *same* category of tail heaviness their range of heavinesses might not overlap, which may seem counterintuitive, but implies once again that only binary tail heaviness comparisons make sense. With regards to PD tail relative tail heaviness, taking the logarithm of the ratio of survival functions simplifies the analysis [69]. The relative terminal tail heaviness of PD versus ED follows from

$$\ln\left[\frac{S_{\mathrm{PD}}(t)}{S_{\mathrm{ED}}(t)}\right] = \alpha \ln\left(\frac{\beta}{t}\right) + \lambda t \quad , \tag{42}$$

where

$$\lim_{t\to\infty}\left[\alpha \ln\left(\frac{\beta}{t}\right) + \lambda t\right] \to \infty \quad . \tag{43}$$

This result is unequivocal; the terminal tail of a PD is always heavier than that of an ED.

## Residence Time

Geometric mean residence time, when robust, can be found using Monte Carlo simulation of a pdf, a tedious process. The median and geometric mean residence times are robust for the GD, PD, exponential and GPC distributions. The harmonic mean residence time ($H$-$MRT$) for PD and GPC are robust, as are the mean residence times ($MRT$) for the exponential and GD distributions. PD and GPC $MRT$ are not defined when $\alpha < 1$, and when $\alpha > 1$, are not very robust. As $\alpha \leq 0.2644$ , their $MRT$ were undefined, but $H$-$MRT_{\mathrm{GPC}}$ were obtained from $\lim_{z\to\beta^+}\left[\int_z^\infty \frac{f(t)}{t}dt\right]^{-1}$ and shown in Table 2. Median-$RT$ occur when the survival functions are one-half, i.e., from Median-$RT = x$; $S(x) = \frac{1}{2}$. The coefficients of variation (CV) were 39.3%

and 45.6% respectively for $H\text{-}MRT_{\text{GPC}}$ and Median-$RT_{\text{GPC}}$. $H\text{-}MRT_{\text{GPC}}$ averaged 0.360 h. Finally, we defined $V_{H\text{-}MRT} = CL_{\text{GPC}}\ H\text{-}MRT$—see Table 7. Dog 1's $V_{H\text{-}MRT}$ is an extreme proportional outlier; its reciprocal being $> 3$ IQR (interquartile range) larger than the third quartile $V_{H\text{-}MRT}$ reciprocal. However, the $V_{H\text{-}MRT}$-values sort best with E2's $V_{SS}$ (Spearman rank correlation coefficient, $rs = 0.96$). Indeed, this agreement is nominally better than the agreement between the two E2 apparent volumes, $V_{SS}$ and $V_{d\text{-}area}$ ($rs = 0.89$, apparent steady-state volume of distribution and terminal apparent volume of distribution, respectively). Although the significance of this is unknown, it would appear that if E2 $V_{SS}$ is measuring something, then GPC's $V_{H\text{-}MRT}$ is as well.

Table 7: Residence time volumes of distribution in (L/kg)

| Dog | GPC $V_{H\text{-}MRT}$ | E2 $V_{SS}$ | E2 $V_{d\text{-}area}$ |
|---|---|---|---|
| 1 | 0.0692 | 4.906 | 21.44 |
| 6 | 0.2059 | 5.782 | 22.66 |
| 5 | 0.3070 | 6.070 | 19.40 |
| 2 | 0.3857 | 6.408 | 24.58 |
| 4 | 0.3865 | 9.071 | 35.54 |
| 7 | 0.4209 | 7.396 | 27.84 |
| 3 | 0.5164 | 9.275 | 37.09 |
| median | 0.3857 | 6.408 | 24.58 |
| IQR | 0.2150 | 3.289 | 14.09 |
| median/IQR | 0.5574 | 0.5133 | 0.5733 |

## Noncompartmental time-sample group, median half-life

Given time-samples, $(t_i, C_i)$ and $(t_{i+1}, C_{i+1})$, the half-life of an exponential connecting them occurs once or more during that time interval, and as per Eq. (5) is

$$\xi = \ln(2)\frac{t_{i+1} - t_i}{\ln(C_i) - \ln(C_{i+1})} \quad ,$$

For subjects having groups of samples drawn at the same times, $t_{i+1} - t_i = \Delta t_i$, the numerator of Eq. (5) is a constant. The $\Delta \ln(C)$ denominator as sampling noise goes to zero is asymptotically a proportional data model—see Eq. (39)—thus is approximately normally distributed. In the limit as $\Delta t \to 0$, the signal reduces in the denominator to an asymptotic mean of 0 leaving constant noise with standard deviation, $k$. Thus, Eq. (5) is a constant times the reciprocal of a normally distributed random variable, which is not the same as the reciprocal of the normal distribution pdf. Rather we substitute $\frac{1}{x} = z$ in $\mathcal{N}_z(0,\, k)$ to yield,

$$\frac{1}{X} \sim \mathcal{N}_{\frac{1}{x}}(0,\, k) = \frac{e^{-\dfrac{1}{2k^2x^2}}}{\sqrt{2\pi}kx^2} \,. \tag{44}$$

which is a special case of the reciprocal normal distribution. For Eq. (5), an attempted mean calculation becomes more erratic when more subjects are included because Eq. (44)'s mean and variance do not exist. This is well known and its tails using Relative tail heaviness methods are Cauchy distribution similar, i.e., $\pm x \to \infty$, $S_{\mathcal{N}_{1/x}(0,k)}(x)/S_{\text{Cauchy}(0,k)}(x) \to \frac{1}{k^2}\sqrt{\frac{\pi}{2}}$.

Although the constant multiplier, $\ln(2)\Delta t$, of Eq. (5) gets smaller as $\Delta t \to 0$, the behaviour of Eq. (44) does not improve (its variance does not exist). $\mathcal{N}_{1/x}(0,\, k)$ is a bimodal distribution with left and right sided fat-tails for which the median measures centrality as $\text{CDF}_{\mathcal{N}_{1/x}(0,\,k)}\,(x=0) = \frac{1}{2}$. Thus, one can use the median values of the subjects' $t_{1/2}$ (include negative ones) from Eq. (5) to plot $t_{1/2}$ versus sample times. However, the mean is pathologic, i.e., $\frac{1}{n}\Sigma_{i=i}^{n}X_i$ diverges as $n \to \infty$, and should never be used.

A final consideration is what mean time on the interval $t_i$ to $t_{i+1}$ of Eq. (5) to assign a half-life as having occurred. As there are 18 to 21 time intervals for the data here, there is only a small numerical range of $[t_i, t_{i+1}]$ for assigning the time of occurrence of a half-life. As the logarithm of time approximately linearised the half-life functions examined, the geometric mean time, $\text{GM}(t) = \sqrt{t_i \times t_{i+1}}$, was used as the $i^{\text{th}}$ time of $t_{1/2}$.

## Parameter errors from model-based bootstrap

Nelder-Mead, also called the simplex method, is a fast global optimisation search routine, and is a practical choice for regression of the GPC function to data. The simplex method is not easily fooled by multiple local minima and if searching a space build like an ice cube tray, will find the least error point in that space. However, the simplex method does not calculate the gradients, Hessians or Jacobians generated during Levenberg-Marquardt or other local optimisation routines that rely on gradients to direct the regression to a lesser, but not necessarily least-error, fit location. Those matrices are frequently used for generating and reporting estimated propagated error of case-wise pharmacokinetic model parameters.

Another possibility for obtaining case-wise parameters errors is to use model-based bootstrap of regression residuals [71]. The model-based bootstrap procedure randomly resamples residuals with replacement and adds those randomised residuals to the model amplitudes at all the original sample times to create synthetic data and that procedure is then repeated multiple times for each case. The synthetic data are then regressed to yield multiple parameter values for each case from which errors and distribution information not otherwise available are extracted. Because of what is being done, we required normality of residual magnitude. The GPC residual values were approximately normally distributed (Results and Figure 3). The E2 residuals were non-normal (Results and Figure 4). The other requirement for model-based bootstrap is that the residuals are relatively unstructured as a list sorted by earliest time of acquisition. We measured the residuals' structure from the data used for Figures 3 and 4 using analysis of variance (ANOVA).[11] ANOVA of residuals with their sample group average values as the test model yielded an adjusted $\text{R}^2_{adj} = 0.1407$ for GPC

[11]ANOVA applied to sample groups provided a population summary of residual variance, rather than case-wise variances as would be a more typical ANOVA application.

models and $R^2_{adj} = 0.5826$ for E2 models. That is, the E2 models had 4.1 times more explained fraction, i.e., structured variance, than GPC models. Thus, model-based bootstrap E2 parameter errors would likely be inaccurate and were not calculated, and for that problem a method that conserves list-wise, i.e., sequence, dependent non-normal loss functions during nonlinear regression would be more appropriate.

Thirty-five GPC simulations were performed and were consistency checked. The bootstrap fit errors ranged from 4.35% to 15.8% rrms% and were not significantly differently distributed from the fit errors from the original data ($p = 0.51$, Cramér−von Mises, two-sample test), and were approximately log-normally distributed ($p = 0.81,\ n = 35$, Cramér−von Mises, one-sample test) with a bootstrap GM[12] of 7.5% and original solution GM of 8.2%, implying that the bootstrap distribution was not significantly inaccurate. The largest value, 15.8%, was a solitary near outlier.[13] This outlier was not implausible for its log-normal distribution (binomial $p = 0.47$ of at least one value $\geq 15.8\%$).

The trial with second largest fit error is illustrated in Figure 12. This photogenic simulation was selected to illustrate the results of the process of simulated data creation. Visually, the original and simulated data appear to have similarly large noise, and the large noise permits good visual separation of the original data and simulated data.

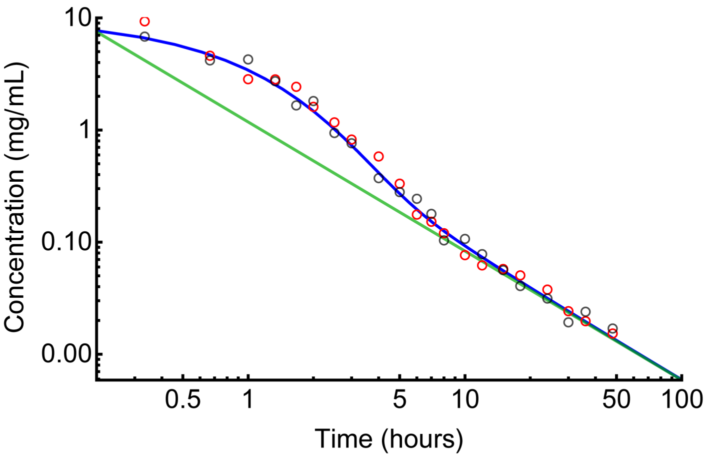


Figure 12: This simulation (dog 4 simulation 17) had a GPC fit error of 14.0%, the second worst fit error in the series of 35 simulations. The original data are shown as red circles, and the bootstrap resampling of residuals are shown as black circles. The blue curve is the GPC simulation model fit, and the green line is the limiting Pareto distribution tail.

The 35 model-based GPC simulations, i.e., only 5 per case, were performed as currently limited by the long runtime for each model solution. That is insufficient to establish precise CV% for each case, but as the ratio between gradient based error propagation results and

[12]The GM (geometric mean) of a lognormal distribution parameterised as $\mathcal{N}_{\ln(x)}(\mu, \sigma)$ is its location, which is also its median value because logarithm is a monotone transform of quantiles.

[13]Outliers are usually defined in terms of the first and third quartiles (Q1, Q3) and the interquartile range (IQR = Q3 − Q1). A near outlier is $<$ Q1 − 1.5 IQR or $>$ Q3 + 1.5 IQR, but is not so extreme as to be a far outlier. Outliers for a normal distribution occur for $\text{erfc}\left[4\,\text{erfc}^{-1}\left(\frac{1}{2}\right)\right]$ of the realisations. Far outliers are $<$ Q1 − 3 IQR or $>$ Q3 + 3 IQR and occur for $\text{erfc}\left[7\,\text{erfc}^{-1}\left(\frac{1}{2}\right)\right]$, where erfc is the complimentary error function.

bootstrap is not unusually a factor of two larger or smaller [72], our results were sufficiently precise to roughly gauge CV% magnitude. At the best of times, individual parameters errors do not represent enough information to fully characterise those parameters and for constrained parameters do not contribute useful information. Additional and more precise quality assurance information arose from the 35 bootstrap parameter distribution statistics for all seven cases, which is more relevant for the examination of outliers and robustness of parameters. Table 8 summarises the GPC regression model parameter results from the simulations with all 35 sets of parameter results from bootstrap as well as screening parametric and nonparametric analysis including 95% confidence intervals for mean and median listed in worksheet *bootstrap results* .xlsx file labelled Supplementary Materials 1.

Table 8: Mean case-wise CV% of GPC parameters, near outliers and parameter ranges for 35 simulations.

| dog | $a$% | $b$% | $\alpha$% | $\beta$ * | $AUC$% | $CL$%† | Peak% |
|---|---|---|---|---|---|---|---|
| 1 | 8.09 | 4.84 | 5.13 | 4↓ 1↑ | 6.22 | 6.42 | 8.98 |
| 2 | 10.5 | 6.89 | 4.90 | 3↓ 2↑ | 1.09 | 1.09 | 46.9 |
| 3 | 8.67 | 5.95 | 3.81 | 3↓ 2↑ | 3.77 | 3.72 | 44.2 |
| 4 | 22.3 | 15.9 | 9.80 | 2↓ 2↔ 1↑ | 5.65 | 5.89 | 78.7 |
| 5 | 20.6 | 11.4 | 6.11 | 3↓ 2↑ | 5.37 | 5.14 | 59.5 |
| 6 | 7.42 | 4.93 | 5.85 | 1↓ 2↔ 2↑ | 2.15 | 2.10 | 8.90 |
| 7 | 17.7 | 20.3 | 5.82 | 1↔ 4↑ | 2.31 | 2.34 | 77.6 |
| rrms% | 14.8 | 11.5 | 6.17 | * | 4.22 | 4.26 | 53.5 |
| all%‡ | 29.7 | 25.0 | 25.9 | * | 28.8 | 39.5 | 81.3 |
| outliers§ | 0 | 0 | 2 | * | 0 | 4 | 1 |
| units | none | per h | none | 5 | $\frac{\text{mg}\cdot\text{h}}{\text{L}}$ | $\frac{D}{AUC}$ | min |
| minimum | 0.265 | 0.557 | 0.117 | 16/35 | 11.4 | 0.515 | 0.563 |
| mean | 0.591 | 0.874 | 0.183 | * | 24.0 | 0.899 | 2.68 |
| median | 0.607 | 0.902 | 0.183 | 27.0 s | 26.1 | 0.747 | 2.02 |
| maximum | 0.898 | 1.37 | 0.295 | 14/35 | 35.5 | 1.71 | 9.13 |

*$\beta$ was constrained and the 5 values per case were either at the lower boundary of 25 s (↓), within the 25—30 s window (↔), or at the 30 s upper boundary (↑).

†$CL$ is L/hr in this table to emphasise $CL = D/AUC$.

‡All 35 values have a CV%, which is larger than the combined CV% (= rrms%) of each case. (Average CV% would be an underestimate.)

§Number of upper near outliers from $n = 35$ simulations.

In this table, the minimum and maximum values correspond to the nonparametric 94.4% confidence intervals for values with $n = 35$ (Weibull method). The rrms% of case-wise CV% values were used to calculate the global case-wise processing errors of parameters, and the CV% values of all 35 simulations were the population deviations for parameters. Note

that the rrms% of individual case parameters CV% were only a fraction of the total CV% for all simulations and all parameters, i.e., the population of dogs had more variability of parameters than the expected case-wise parameter errors. $\beta$ was a constrained parameter. Its values were located in a 16, 5, and 14 split between, respectively, being at the lower constraint boundary of 25 s, between the 25 to 30 s boundaries, and at the 30 s upper boundary. Even if that fairly even 16/14 split at the constraint boundaries were due to noise, it would be suspiciously unbiased noise. For example, if all the values were grouped at the lower constraint boundary of 25 s, one would have reason to suspect that a better regression answer would occur at some constraint value less than 25 s. The fact that 5 of the 35 $\beta$-values occurred between 25 and 30 is not inconsistent with having set plausible constraints, and that despite not having primary study $\beta$-values in that time window. This is the physical equivalent of hitting a one-inch wide, elongated strip of masking tape with 5 darts of 35 thrown from 20 feet away. The most variable parameter in the table is the (approximately lognormal distributed) time of peak concentration, which varied from 0.56 to 9.1 min but had only a single (i.e., not implausible) near outlier. That variability did not adversely affect $AUC$-values as those latter were of narrow range with no outliers, had low CV% values and insignificantly ($p = 0.036, n = 35$) negative correlation with peak time. Both times of peak concentration and $\beta$ first peripheral venous arrival times are unobserved events predicted by the GPC model whose variability would be better assessed using earliest observations available sooner than the 20 min time-samples in the data set used.

As it goes to robustness, the examination of outliers is important. For a normal distribution near outliers occur for 0.698% of the realisations, whereas far outliers occur for only 0.000234%.[13] Four of the seven (upper) near outliers were from $CL$, which inherited a heavier than normal right tail from reciprocation because $CL = D/AUC$, and $AUC$ as well as $1/CL$ ($= AUC/D$) were more normally distributed with no near outliers. The other three near outliers were similarly insufficient to suggest any robustness problem, and infrequent near outliers often suggest non-normality, which do not require investigation as erratic values. However, far outliers are so infrequent in the normal case that consideration should be given to pathologic or extreme non-normality of a parameter, to effects arising from some inappropriate quality of the data itself, and/or to some systemic methodological problem. The outliers listed in the table were near outliers, were few in number and were all upper range suggesting heaviness of right tails for some parameters, and no far outliers to suggest a lack of robustness of the methodology were seen.
